# Signaling pathways and potential therapeutic targets in acute respiratory distress syndrome (ARDS)

**DOI:** 10.1186/s12931-024-02678-5

**Published:** 2024-01-13

**Authors:** Qianrui Huang, Yue Le, Shusheng Li, Yi Bian

**Affiliations:** 1 Department of Critical Care Medicine, Tongji Hospital, Tongji Medical College, Huazhong University of Science and Technology, No.1095, Jie Fang Avenue, Wuhan, 430030 China; 2 Department of Emergency Medicine, Tongji Hospital, Tongji Medical College, Huazhong University of Science and Technology, No. 1095, Jie Fang Avenue, Wuhan, 430030 China; 3 Department of Critical Care Medicine, Zhongda Hospital, School of Medicine, Southeast University, 87 Dingjia Bridge, Hunan Road, Gu Lou District, Nanjing, 210009 China

**Keywords:** Acute respiratory distress syndrome, Pathogenesis, Pathophysiology, Signaling pathways, Therapeutics

## Abstract

Acute respiratory distress syndrome (ARDS) is a common condition associated with critically ill patients, characterized by bilateral chest radiographical opacities with refractory hypoxemia due to noncardiogenic pulmonary edema. Despite significant advances, the mortality of ARDS remains unacceptably high, and there are still no effective targeted pharmacotherapeutic agents. With the outbreak of coronavirus disease 19 worldwide, the mortality of ARDS has increased correspondingly. Comprehending the pathophysiology and the underlying molecular mechanisms of ARDS may thus be essential to developing effective therapeutic strategies and reducing mortality. To facilitate further understanding of its pathogenesis and exploring novel therapeutics, this review provides comprehensive information of ARDS from pathophysiology to molecular mechanisms and presents targeted therapeutics. We first describe the pathogenesis and pathophysiology of ARDS that involve dysregulated inflammation, alveolar-capillary barrier dysfunction, impaired alveolar fluid clearance and oxidative stress. Next, we summarize the molecular mechanisms and signaling pathways related to the above four aspects of ARDS pathophysiology, along with the latest research progress. Finally, we discuss the emerging therapeutic strategies that show exciting promise in ARDS, including several pharmacologic therapies, microRNA-based therapies and mesenchymal stromal cell therapies, highlighting the pathophysiological basis and the influences on signal transduction pathways for their use.

## Background

Acute Respiratory Distress Syndrome (ARDS) is a clinical syndrome characterized by severe continuous hypoxemia, which can be caused by intrapulmonary and/or extrapulmonary causes. The most common cause is pneumonia, especially bacterial and viral pneumonia. In terms of extrapulmonary factors, sepsis due to non-pulmonary sources is the most common cause of ARDS. ARDS is mainly characterized by diffuse alveolar injury, including excessive inflammation, increased epithelial and vascular permeability, alveolar edema, and hyaline membrane formation. Although there are many pieces of research on the pathogenesis of ARDS, few specific pharmacotherapies for this disease can be used clinically [1]. Treatment of ARDS is generally supportive with lung-protective mechanical ventilation. Thus, the mortality of ARDS remains unacceptably high. The latest data from the Large Observational Study to Understand the Global Impact of Severe Acute Respiratory Failure study reports 40% hospital mortality of ARDS [2]. The outbreak of coronavirus disease 2019 (COVID-19) worldwide caused by severe acute respiratory syndrome coronavirus 2 has correspondingly increased the mortality of ARDS, leading to devastating economic and medical burden worldwide.

Recently, multiple studies have been published on the molecular mechanisms involved in pathogenesis and pathophysiology of ARDS and have made substantial progress. Some potential pharmacotherapeutic agents have proven efficacy in preclinical models of ARDS by targeting specific molecules or regulating related signal pathways. Considering the complex pathophysiology of ARDS characterized by inflammation-mediated disruptions in alveolar-capillary permeability, reduced alveolar fluid clearance (AFC), and oxidative stress, a comprehensive understanding of the underlying signal transduction within the pathogenesis and pathophysiology of ARDS significantly offers deep insight into development and progress of ARDS. This helps to provide a theoretical foundation and motivation for the discovery of novel therapeutic strategies to treat ARDS. In this review, we outline the available literature on mechanisms of pathophysiology and signal transduction for ARDS. Both novel and canonical signal transduction pathways are summarized and functionally classified according to their pathophysiological roles in ARDS, including inflammation, increased alveolar-capillary permeability, reduced AFC, and oxidative stress. We highlight these pathophysiological mechanisms by presenting the location and effects of the underlying signaling pathways in tissue, cells, and organelles. Furthermore, we introduce the recent findings of potential therapeutic strategies agents that target specific signaling pathways to modulate the above four aspects of pathophysiological mechanisms of ARDS.

## Pathophysiology and pathogenesis of ARDS

The normal lung functions to facilitate oxygen transfer and carbon dioxide excretion, a process established by the alveolar–capillary unit. The pulmonary endothelium consists of a monolayer of endothelial cells linked by adherens junctions and tight junctions. It contributes significantly to the precise regulation of fluid and solutes to prevent lung flooding [3]. The alveolar epithelium is lined by alveolar type I cells, which form a tight barrier allowing gas exchange, and alveolar type II cells, responsible for producing surfactant to reduce surface tension and keep alveoli open. Both types of cells can absorb edema fluid from the alveolar space that help oedema resolution. The composition of the normal alveolus also includes alveolar macrophages (AMs), which provide host defense [4].

**Fig. 1:**
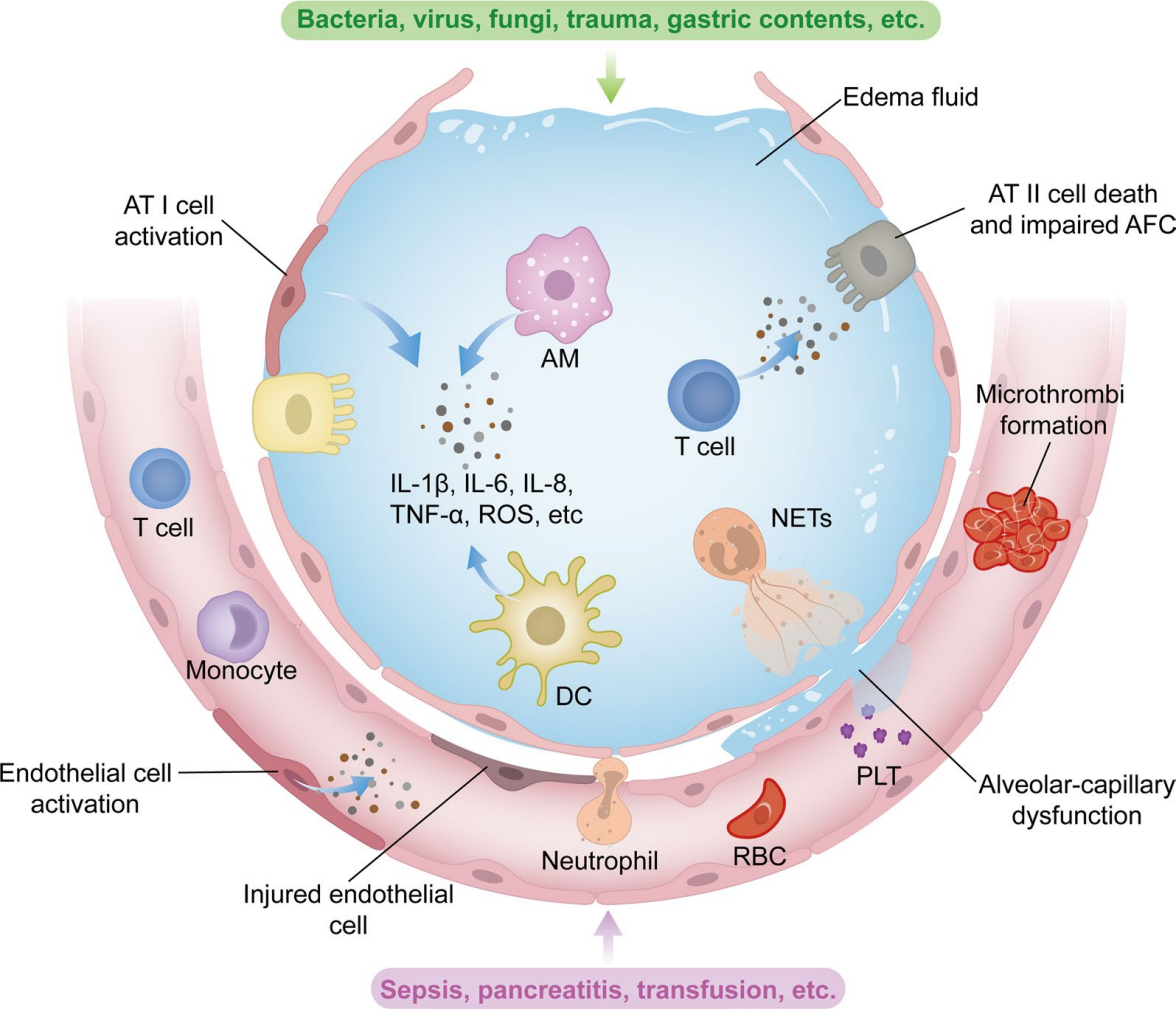
Pathophysiology of ARDS. The pathophysiology of ARDS is complex, involving dysregulation of inflammation, alveolar-capillary injury, impaired alveolar fluid clearance and oxidative stress. In case of pulmonary causes such as pneumonia or aspiration, the alveolar epithelium can be directly injured or affected by inducing inflammation. Activation of alveolar epithelial cells, resident alveolar macrophages AMs and dendritic cells DCs leads to the production of inflammatory cytokines and chemokines, recruiting circulating innate and adaptive immune cells into the airspaces. These immune cells amplify inflammation by releasing additional inflammatory molecules. Neutrophils, upon migration, release cytotoxic factors such as ROS and NETs, contributing to the disruption of alveolar-capillary barrier. Endothelial injury due to inflammation activates procoagulant pathways and results in microthrombi formation. These effects ultimately lead to alveolar–capillary barrier injury, allowing the leakage of protein-rich fluid from vasculature into interstitial space and alveoli. The filling of airspaces with edema fluid causes hypoxemia and hypercapnia, which, in turn, reduce fluid and ion clearance. In cases of extrapulmonary ARDS, lung injury is derived from pulmonary endothelial cells. Similarly, pulmonary endothelial dysfunction triggered by circulating injurious molecules can induce inflammatory injury toward the alveolar epithelium, resulting in increased permeability and alveolar oedema. AFC, alveolar fluid clearance; AM, alveolar macrophage; AT I, alveolar type I cell; AT II, alveolar type II cell; DC, dendritic cell; IL-1β, interleukin-1β; IL-6, interleukin-6; IL-8, interleukin-8; NETs, neutrophil extracellular traps; PLT, platelet; RBC, red blood cell; ROS, reactive oxygen species; TNF-α, tumor necrosis factor-α

Regardless of the primary disease, the pathophysiologic manifestations of ARDS are very similar. Essentially, these syndromes reflect severe injury resulting in dysfunction of the alveolar-capillary barrier, impaired AFC, and oxidative injury due to unregulated acute inflammatory responses (Fig. 1).

### Excessive inflammation

Acute lung injury (ALI) is initially caused by inflammation, which is mediated by an intricate interplay of inflammatory cytokines and chemokines released by various cell types in the lungs [5]. In response to direct insults such as bacteria, viruses and gastric contents, the pattern recognition receptors (PRRs) expressed in innate immune cells in alveolar, such as AMs, alveolar epithelial cells (AECs), and dendritic cells (DCs) are initially activated [6]. They release inflammatory cytokines to amplify immune response by acting locally on other cells and recruiting circulating immune cells into the airspace. This effect further induces amplification of inflammation and aggravates lung injury [7]. Neutrophils have been widely implicated in playing a critical role in the pathogenesis of ARDS. Activation of accumulated neutrophils in the alveolar space and lung microvasculature produces numerous cytotoxic substances, including granular enzymes, pro-inflammatory cytokines, and neutrophil extracellular traps (NETs), resulting in sustained inflammation and alveolar-capillary barrier injury [8]. Additionally, the influx of adaptive immune cells also plays an essential role in promoting inflammatory injury and thrombosis by producing various cytotoxic molecules such as cytokines, perforin, granzyme B, and autoantibodies [9–11].

Different from intrapulmonary ARDS, where alveolar inflammation occurs initially, inflammatory injury caused by indirect factors is driven from systemic compartment and spreads towards the alveolar compartment [12]. Lung endothelium activation, triggered by circulating stimuli released from extrapulmonary lesions into the blood, can also produce proinflammatory molecules to facilitate the adherence and infiltration of immune cells, further leading to vascular inflammation and alveolar damage [13].

Unlike other organs, the lung is continually exposed to various environmental challenges, including microbial pathogens, pollution, dust, and more [14]. Similarly, pulmonary endothelial cells are exposed to circulating inflammatory components, hormones, exotoxins, and endotoxins, which interact with both local and systemic inflammatory responses [15]. The alveolar epithelium, lung endothelium, and the cross-talk within the immune system collectively constitute the physical barrier and immune homeostasis in the lung [16]. Traditionally, a systemic inflammatory cascade has been used to describe immune dysregulation during ARDS, but this perspective has been challenged by the recognition of immune compartmentalization response [17]. It was previously observed that intratracheal administration of lipopolysaccharide (LPS) leads to a significant increase in tumor necrosis factor-α (TNF-α) levels in bronchoalveolar lavage fluid (BALF) but not in plasma, whereas intravenous LPS administration results in a potential increase in TNF-α levels in blood but not in BALF [14]. Recently, the compartmentalization of inflammation specific to the lung has also been observed in COVID-19-related ARDS [18]. Hence, when exploring biomarkers for diagnosis and subphenotyping, as well as investigating the pathophysiology and signaling pathways of ARDS, it is important to consider this organ-specific immune compartmentalization.

### Endothelial and epithelial permeability

Another core pathophysiologic derangement is the increased permeability of two separate barriers, the lung endothelium and alveolar epithelium. As a result of dysregulated immune response, the impaired endothelial barrier can occur owing to disruption of intercellular junctions, endothelial cell death, and glycocalyx shedding. In normal lungs, maintenance of the endothelial barrier is mediated by vascular endothelial cadherin (VE-cadherin), which tightly connects adjacent endothelial cells, and prevents leucocyte migration and vascular leak [19, 20]. During lung injury, inflammatory factors mediate the phosphorylation of VE-cadherin, resulting in its endocytosis. Endocytosis of VE-cadherin induces gaps between endothelial cells, leading to increased permeability [21]. Moreover, the disruption of endothelial tight junctions, such as reduction in protein levels of occludins and zonula occludens (ZOs), can also promote intercellular permeability [22]. In addition, endothelial cell death can cause increased permeability to proteins and solutes [23].

Similar to endothelial injury, disruptions of epithelial barrier function involve the dissociation of intercellular junctions, primarily E-cadherin junctions, and alveolar epithelial cell death. In ARDS, various damaging factors can ruin the alveolar epithelium directly or by inducing inflammation. The inflammatory injury caused by immune response inevitably aggravates the direct damage to AECs, including cell death and intercellular junction disruption, leading to increased alveolar epithelial permeability [3].

### Alveolar fluid clearance

The failure to absorb alveolar edema fluid significantly contributes to increased mortality in ARDS. Basal AFC is determined by ion and fluid transportation of alveolar epithelium. In normal epithelium, sodium is transported through the apical surface via the epithelial Na^+^ channel (ENaC) and then pumped from the basolateral surface into the lung interstitium by the sodium–potassium adenosine triphosphatase (Na,K-ATPase). while chloride is transported through the cystic fibrosis transmembrane conductance regulator (CFTR) channels [24]. The directional ion transport establishes an osmotic gradient that passively drives the removal of water from the alveoli to the interstitium through aquaporins or intracellular routes [25]. Subsequently, fluid can be eliminated via lymphatic drainage and lung microcirculation [4]. However, these transport systems and functions are impaired in ARDS patients due to epithelium injury caused by elevated levels of proinflammatory cytokines, leading to the loss of ion channels and pumps [26]. Increased permeability of liquid and protein into the alveolar space greatly exceeds the capability of AFC. The alveolar space filled with oedematose fluid decreases diffusion of carbon dioxide and oxygen, thus leading to hypoxia and hypercapnia and further impair AFC by inhibiting the Na,K-ATPase activity or inducing Na,K-ATPase endocytosis [27–29]. Patients with severe hypoxia frequently require mechanical ventilation to facilitate breathing. High tidal volumes and elevated airway pressures can induce biomechanical inflammatory injury and reduce Na,K-ATPase activity [30]. All of these events significantly inhibit AFC, leading to persistent alveolar edema, refractory hypoxemia and/or carbon dioxide retention.

### Oxidative stress and lung injury

Oxidative stress, resulting from the production of reactive oxygen species (ROS), plays an important role in ARDS progression and lung injury. In response to inflammatory stimuli, various cell types in the lung can generate ROS. Most of the damaging ROS produced by innate immune cells like AMs and recruited leukocytes, cause cell injury by inducing oxidation and cross-linking of proteins, lipids, DNA, and carbohydrates [31]. Significantly, ROS produced by neutrophils disrupt the endothelial barrier, facilitating the migration of recruited inflammatory cells across it and thereby aggravating inflammation [32]. Similarly, activated AECs and pulmonary endothelial cells can produce ROS, directly contributing to signaling transduction that increases alveolar-capillary permeability and impairs sodium ion transport, thereby impairing the reabsorption of fluid from the alveolar compartment [33, 34]. In fact, oxidative stress and inflammatory response always reinforce each other in the progression of ARDS. Although there are many checks and balances in this system in the form of antioxidant defenses in ALI/ARDS, an excessive production of ROS overwhelms endogenous antioxidants, leading to oxidative cell injury and exacerbation of inflammatory responses [35].

## Molecular mechanisms and signaling pathways

In this part, we discuss the specific functions of signaling pathways in regulating pathophysiological processes of ARDS including lung inflammation, alveolar-capillary permeability, and AFC, which contributes to discovering the potential and novel therapeutic strategies.

### Signaling pathways related to inflammation

#### Pattern recognition receptors

The innate immune activation in both direct or indirect lung injury is considered a potent driver of lung inflammation. It is triggered by endogenous damage-associated molecular patterns (DAMPs) released by cells under conditions of stress, injury, or cellular death, as well as microbial-derived pathogen-associated molecular patterns (PAMPs). DAMPs and PAMPs can be recognized by PRRs expressed in host cells, initiating PRR-induced signaling pathways that lead to the expression of inflammatory factors. The following section mainly introduces the signaling pathways induced by PRRs, including toll-like receptors (TLRs), nucleotide-binding leucine-rich repeat receptors (NLRs), retinoic acid-inducible gene I (RIG-I) -like receptors (RLRs), cytoplasmic DNA sensors (CDSs), and receptors for advanced glycation end products (RAGEs) in relation to ARDS.

**Fig. 2:**
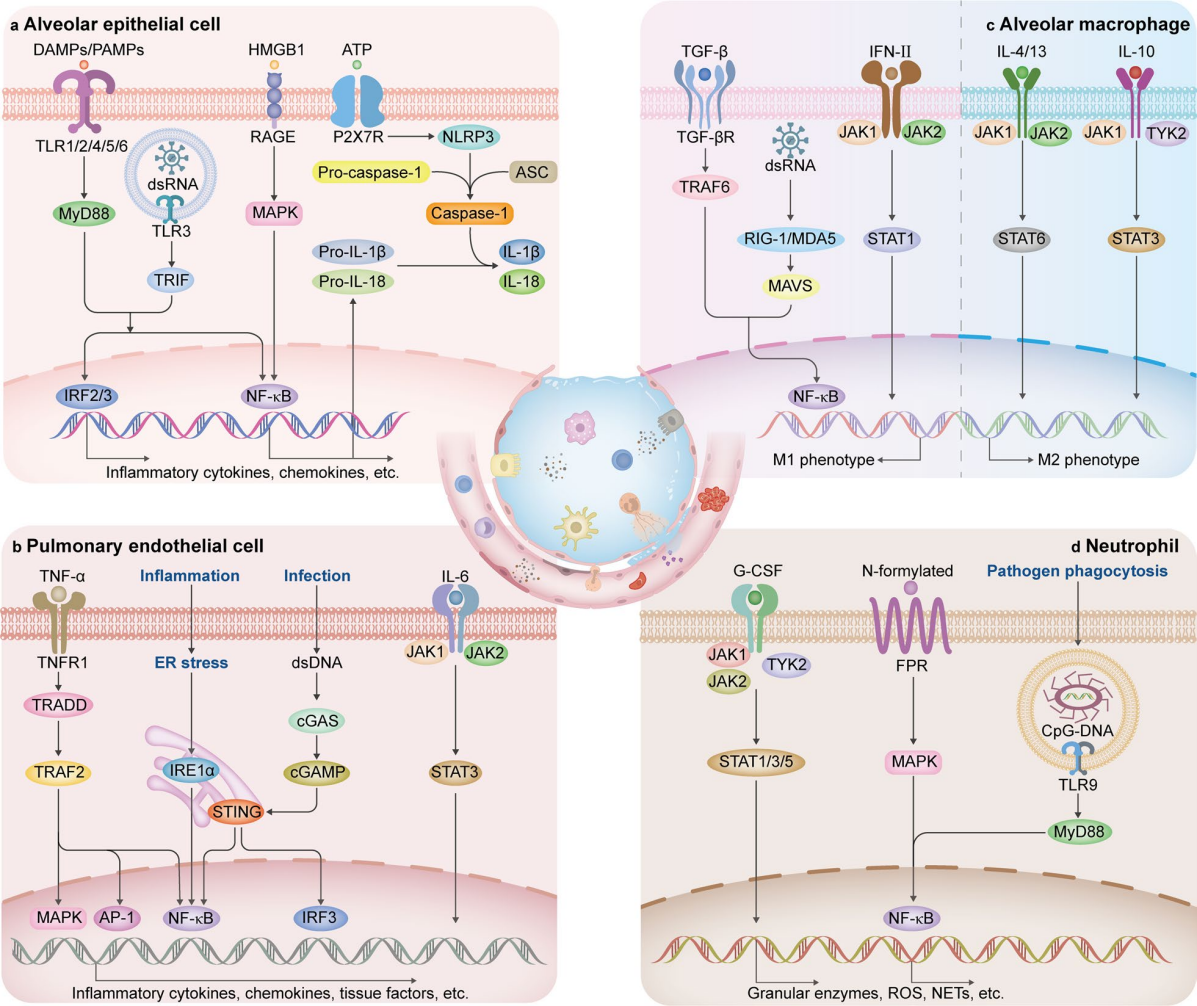
Inflammatory signaling pathways activated in AECs, pulmonary endothelial cells, AMs and neutrophils during ARDS. **a** Signaling pathways in AEC activation, leading to an increase of inflammation. **b** Signaling pathways in pulmonary endothelial cells, promoting inflammation and coagulation. **c** Signaling pathways in the regulation of macrophage polarization. The left area of the dashed line promotes AM to exhibit M1 phenotype, while the right area of the dashed line promotes M2 polarization. **d** Signaling pathways in neutrophil activation. AP-1, activator protein-1; ASC, apoptosis-associated speck-like protein containing a CARD; cGAMP, cyclic dinucleotide cyclic GMP-AMP; cGAS, cyclic GMP-AMP synthase; DAMPs, damage-associated molecular patterns; dsRNA, double-stranded RNA; ER, endoplasmic reticulum; FPR, N-formyl peptide receptor; G-CSF, granulocyte colony-stimulating factor; HMGB1, high-mobility group box 1; IRE1α, inositol requiring kinase 1α; IRF, interferon regulatory factor; JAK, janus kinase; MAPK, mitogen-activated protein kinase; MAVS, mitochondrial antiviral signaling protein; MDA5, melanoma differentiation-associated gene 5; MyD88, myeloid differentiation primary response gene 88; NF-κB, nuclear factor-κB; NLRP3, nucleotide-binding domain leucine-rich repeat protein 3; P2X7R, P2X7 receptor; PAMPs, pathogen-associated molecular patterns; RAGE, receptor for advanced glycation end product; RIG-I, retinoic acid-inducible gene I; STAT, signal transducer and activator of transcription; STING, stimulator of interferon gene; TGF-β, transforming growth factor-β; TGF-βR, transforming growth factor-β receptor; TLR, toll-like receptor; TNFR1, TNF receptor 1; TNF-α, tumor necrosis factor-α; TRADD, TNFR-associated death domain; TRAF, TNFR–associated factor; TRIF, Toll/interleukin-1 receptor-domain-containing adaptor-inducing interferon-β; TYK2, tyrosine kinase 2; IFN, interferon

To date, 10 functional TLRs have been identified in humans. TLR1,2,4,5 and 6 are surface-expressed, while TLR3,7,8 and 9 are located in lysosomal or endosomal membranes. In the lung, different TLRs are expressed in various cell types and recognize specific PAMPs and DAMPs to generate inflammatory signals (Table 1) [6, 36]. The ability of TLRs to activate transcription factors interferon regulatory factors (IRFs) or nuclear factor-κB (NF-κB), requires the recruitment of adaptor proteins, including myeloid differentiation primary response gene 88 (MyD88) and Toll/interleukin-1 (IL-1) receptor-domain-containing adaptor-inducing interferon-β (TRIF). MyD88 is utilized by all TLRs except TLR3, and TRIF is specifically recruited by TLR3 [37]. Activation of IRFs and NF-κB triggered by TLRs are actively involved in the production of type I-interferons (IFNs) and pro-inflammatory cytokines, respectively (Fig. 2a). However, in the context of ARDS, TLR signals are accompanied by an overwhelming production of pro-inflammatory cytokines. Notably, TLRs elicit special responses in polymorphonuclear neutrophil granulocytes to aggravate inflammation during ARDS. Previous studies have revealed that TLR9 and TLR4 contribute to the release of NETs, which contain DAMPs of proteases, histones, and self-DNA to induce inflammation and thrombi development (Fig. 2d) [37–40]. A recent finding indicates that TLR9 activation induces neutrophil elastase and proteinase 3-mediated shedding of the complement component 5a receptor, resulting in a decreased ability to clear bacteria and prolonged ALI in mice [41]. Interestingly, the activation of TLR4 has been shown to play a dual role in regulating lung inflammation. TLR4 of AMs activated by heat shock protein (HSP) 70 conditionally promotes clearance of apoptotic neutrophils by preventing a disintegrin and metalloprotease 17 -mediated cleavage of Mer receptor tyrosine kinase, thereby promoting the outcome of ventilator-induced lung injury (VILI) [42]. Therefore, precise regulation of TLR signals to suppress inflammation and promote the resolution of ARDS may be an effective strategy.

**Table 1:** ARDS related PRRs and non-PRRs, their lung tissue distribution and recognition of PAMPs and DAMPs

Family	Member	Distribution	PAMPs	DAMPs
TLRs	TLR1	AECs, AMs, neutrophils	Triacyl lipopeptides	?
	TLR2	AECs, AMs, pulmonary endothelial cells, neutrophils	Diacyl lipopeptides, lipoteichoic acids	Histones, HMGB1, oxidized phospholipids
	TLR3	AECs	Viral dsRNA	Messenger RNA
	TLR4	AECs, AMs, neutrophils, pulmonary endothelial cells	LPS	Fibrinogen, HMGB1, hyaluronan, oxidized lipoproteins, phospholipids, histones, heat shock proteins
	TLR5	AECs, neutrophils	Flagellin	?
	TLR6	AECs, AMs, neutrophils	Bacterial lipopeptides	?
	TLR7	AECs, DCs, neutrophils	Microbial ssRNA	Host DNA fragments, microRNAs
	TLR8	AECs, neutrophils	Microbial ssRNA	Host DNA fragments, microRNAs
	TLR9	AECs, DCs, neutrophils	Microbial CpG-DNA	Host DNA fragments, microRNAs, mitochondrial CpG-DNA
NLRs	NOD1	Widely expressed	Peptidoglycan	?
	NOD2	AECs, leukocytes	Muramyl dipeptide, ssRNA	?
	NLRP1	AECs, leukocytes	Muramyl dipeptide	?
	NLRP3	AECs, immune cells, pulmonary endothelial cells	Microbial pore-forming toxins, microbial DNA or RNA, muramyl dipeptide	ATP, Ca^2+^ influx, hyaluronan, K^+^ efflux, oxidized mitochondrial DNA, ROS, uric acid,
	NLRC4	AMs	Flagellin	?
	NAIP	AECs, AMs	Flagellin	?
RLRs	RIG-I	Widely expressed	5’ triphosphate RNA	?
	MDA5	Widely expressed	5’ triphosphate RNA	?
CDSs	cGAS	Widely expressed	Microbial dsDNA	Self-DNA
	AIM2	Innate immune cells	Microbial dsDNA	Self-DNA, NETs
RAGE	RAGE	Widely expressed	LPS, microbial DNA, viral and parasitic proteins	HMGB1, S100 family
Non-PRRs	P2X7	Alveolar type I cells	–	ATP
	TRP channels	AECs, immune cells, pulmonary endothelial cells	Environmental irritants	Environmental irritants
	FPR	AECs, neutrophils	N-formylated peptides	N-formylated peptides

“?” in the table indicates the absence of information regarding the corresponding DAMPs or PAMPs recognized by the receptors, and “–” indicates no available information

AECs, alveolar epithelial cells; AIM2, absent in melanoma 2; AMs, alveolar macrophages; CDCs, cytoplasmic DNA sensors; cGAS, cyclic GMP-AMP synthase; DAMPs, damage-associated molecular patterns; DCs, dendritic cells; dsRNA, double-stranded RNA; FPR, N-formyl peptide receptor; HMGB1, high-mobility group box 1; LPS, lipopolysaccharide; MDA5, melanoma differentiation-associated gene 5; NAIP, neuronal apoptosis inhibitory protein; NETs, neutrophil extracellular traps; NLRs, nucleotide-binding leucine-rich repeat receptors; NLRC4, nucleotide-binding oligomerization domain like receptor subfamily C 4; NLRP, nucleotide-binding domain leucine-rich repeat protein; NOD, nucleotide-binding oligomerization domain; PAMPs, pathogen-associated molecular patterns; RAGE, receptor for advanced glycation end product; RIG-I, retinoic acid-inducible gene-I; RLRs, RIG-like receptors; ROS, reactive oxygen species; TLR, toll-like receptor; TRP, transient receptor potential

NLRs are also the commonly studied innate immune system receptors involved in ARDS. NLRs are cytoplasmic PRRs, which can be divided into different subfamilies according to their N-terminal domains, including nucleotide-binding oligomerization domain (NOD), nucleotide-binding domain leucine-rich repeat protein (NLRP), neuronal apoptosis inhibitory protein (NAIP), and nucleotide-binding oligomerization domain like receptor subfamily C (NLRC) [6, 43]. The NOD1 and NOD2 mainly detect bacterial components, recruiting downstream receptor-interacting-serine/threonine-protein kinase 2, which leads to NF-κB or mitogen-activated protein kinase (MAPK) activation [36]. The NLRP1, NLRP3, NLRC4 and NAIP have been characterized to assemble inflammasomes in the lung (Table 1) [6]. In general, the activation of the inflammasome requires two independent signals. The priming signal is the upregulation of NLRs, pro-IL-1β, pro-IL-18, and pro-caspase-1 through NF-κB activation. The second step is induced by NLRs responding to a variety of PAMPs and DAMPs inside the cell (Table 1) [44]. The activated NLRs further assemble inflammasomes to mediate caspase-1-dependent cleavage of pro-IL-1β and pro-IL-18. The secretion of mature forms of IL-1β and IL-18 can further induce inflammation by recognizing their respective cytokine receptors to trigger MyD88/NF-κB signaling [45]. In addition, inflammasome activation can induce cell pyroptosis through caspase-1-mediated proteolysis of gasdermin D, resulting in the formation of pores on the cell membrane and subsequent cell rupture [46]. Pyroptosis leads to a large release of DAMPs and inflammatory mediators (incl. IL-1β and IL-18) that enhances further inflammatory responses [45]. The synergistic interaction between NF-κB and NLRs may account for the supranormal release of cytokines. Thus, inhibition of NF-κB signal and targeting NLRs appear to hold potential for mitigating inflammasome-induced ARDS and the subsequent cytokine storm.

The surveillance of abnormal nucleic acids from invading pathogens or damaged cells is conducted by PRRs including RIG-I, melanoma differentiation-associated gene 5 (MDA5), cyclic GMP-AMP synthase (cGAS), and absent in melanoma 2 (AIM2). Both the cytosolic receptor RIG-I and MDA5 expressed in various host cells belong to RLRs, which provide important defense against viral infections (Table 1) [47, 48]. They recognize viral RNA containing a 5′-triphosphate end and subsequently activate the downstream adapter mitochondrial antiviral signaling protein (MAVS) to induce the activation of IRFs and NF-κB (Fig. 2c) [49]. These signals result in the expression of antiviral type I-IFNs and other inflammatory cytokines [50]. However, there is evidence that RLR signaling cascades induce excess inflammation in ARDS, clinically manifested by upregulation of inflammatory cytokines in the bronchoalveolar lavage fluid of patients with severe viral infections [51].

The abnormal presence of DNA in cytoplasm either from infection or cellular damage induces immune responses through the cytoplasmic DNA sensors, including cGAS and AIM2 (Table 1). cGAS binds to double-stranded DNA, driving the synthesis of cyclic dinucleotide cyclic GMP-AMP (cGAMP), which activates the stimulator of the interferon gene (STING), an endoplasmic reticulum (ER) membrane protein. Activated STING induces the production of inflammatory factors through the activation of downstream NF-κB and IRF 3 (Fig. 2b) [52]. AIM2 detects double-stranded DNA to assemble an AIM2 inflammasome complex, which contains AIM2, apoptosis-associated speck-like protein containing a CARD (ASC), and caspase-1. This complex regulates the maturation of IL-1β and IL-18, as well as induces cell pyroptosis [43, 53]. Similarly, sustained activation of these pathways is detrimental to the host. For instance, self-DNA released by cell death or cellular stress after severe acute respiratory syndrome coronavirus 2 (SARS-CoV-2) infection may activate the cGAS-STING pathway, leading to excessive production of inflammatory factors and exacerbating the severity of COVID-19 [52]. Of note, the cGAS-STING signaling pathway has been shown to phosphorylate the signal transducer and activator of transcription (STAT) 1, which results in the production of adhesion molecules and chemokines to promote immune cell adhesion and migration during ARDS in vivo [54]. Thus, targeting signaling pathways associated with nucleic acid sensors offers potential avenues for anti-inflammatory therapy in ARDS.

RAGE is a PRR highly expressed in the lungs, particularly in AECs (Table 1) [55]. It exists in two forms: membrane-bound and soluble. Membrane-bound RAGE can recognize a variety of ligands (Table 1), triggering various intracellular cascades including NF-κB, MAPK, and phosphatidylinositol 3-kinase (PI3K)/protein kinase B (AKT), ultimately leading to the induction of inflammatory factors (Fig. 2a) [56]. Since the expression of RAGE is significantly upregulated during ARDS, persistent inflammation from RAGE activation may induce harmful effects [57]. In contrast, soluble RAGE is thought to be protective, as it retains the ability of ligand binding while lacking signaling function [58]. Together, targeting membrane-bound RAGE to inhibit inflammatory signaling pathways or competitive binding RAGE ligands through the administration of soluble RAGE could be a therapeutic strategy for ARDS.

In addition to PRRs, there are other receptors that serve as positive regulators of inflammation by recognizing various DAMPs or PAMPs (Table 1). The purinergic ionotropic receptors P2X7 are membrane ion channels involved in the activation of NLRP3 inflammasomes by recognizing extracellular ATP (Fig. 2a) [59, 60]. The transient receptor potential (TRP) channels on the cell surface allow Ca^2+^ influx to initiate the NF-κB-dependent inflammatory responses, which can be triggered by environmental irritants such as inflammatory cytokines and pathogens [61–63]. The N-formyl peptide receptor (FPR) is a member of G-protein-coupled receptors (GPCRs). It recognizes N-formylated peptides derived from bacterial or mitochondria, activating downstream MAPKs, AKT, and NF-κB pathways to induce inflammation [64]. Activation of neutrophils in response to the FPR signal leads to inflammatory responses, such as elastase release, oxidative burst, and chemotactic migration (Fig. 2d) [65]. The activation of these signaling pathways significantly contributes to the development of robust inflammatory responses during ARDS. Conversely, adenosine receptors, which also belong to GPCRs, have been reported for their advantageous anti-inflammatory effects in ARDS. Notably, there is an essential link between hypoxia and inflammatory signaling, serving as a vital physiological protective mechanism to alleviate acute lung inflammation [66]. Mechanistically, cytoplasmic hypoxia-inducible factors (HIFs) stabilize in response to hypoxia and translocate to the nucleus to induce the transcription of adenosine receptors. Besides, the increased release of extracellular ATP/ADP during inflammation also raises the adenosine levels, which promotes a feedback loop that attenuates inflammation [67, 68]. A subtype of adenosine receptors, the A2A receptor, has been identified as the target gene of HIF-1α in alveolar epithelium contributes to lung protection during ALI [69]. In addition, Ko et al. found that A2A receptor exerts anti-inflammatory functions by inhibiting downstream MAPK and NF-κB [70]. However, the physiological protecting HIF/adenosine signaling is often compromised in ARDS patients due to the necessity of hyperoxic conditions in the lung, which may exacerbate acute inflammatory lung injury [71].

#### NF-κB signaling pathway

NF-κB is a transcription factor named for its specific binding to a conserved sequence in the nuclei of activated B lymphocytes [72]. In a resting state, NF-κB exhibits no transcriptional activity as it binds to the NF-κB inhibitor (IκB) in the cytoplasm. Upon activation of the IκB kinase by upstream signals, it induces the dissociation and subsequent degradation of IκB protein from NF-κB. Consequently, NF-κB translocates to specific DNA target sites in the nucleus, initiating the transcription and expression of inflammatory genes [73]. The NF-κB signal can be triggered by multiple stimuli, including cytokines (e.g., TNF-α and IL-1β), microbial infection (LPS), activated PRRs as described above, stress (e.g., ER stress and ROS), as well as elevated CO_2_ during hypercapnia [74, 75]. Notably, aberrant regulation of NF-κB is implicated to induce detrimental inflammation in ARDS [76]. The NF-κB pathway produces a variety of cytokines, chemokines and adhesion molecules, contributing to processes encompassing inflammation, immune cell recruitment, cell adhesion and cell differentiation. For instance, after the exposure of the lungs to noxious agents, NF-κB activation can be initiated by PRRs in both epithelial and endothelial cells, as well as resident immune cells, primarily AMs. Subsequently, these activated cells release cytokines (such as TNF-α and IL-1β) and chemokines (such as IL-8 and monocyte chemoattractant protein-1), amplifying inflammation by activating adjacent cells and recruiting additional immune cells from the peripheral tissues [74]. NF-κB activation also promotes AM polarization into classically activated (M1) macrophages, which overproduce cytokines to drive cytokine storm [77]. In the endothelium, activated NF-κB triggers the expression of adhesion molecules, which promotes the recruited immune cells to adhere and cross the alveolar-capillary barrier to reach the alveolar space, where they propagate inflammation and injury through the production of sustained inflammatory cytokines [78]. Besides, NF-κB can downregulate the anticoagulation proteins to cause intravascular coagulation and thrombin generation, which in turn aggravates inflammatory lung injury [79]. All of these events together contribute to the progression of ARDS. Therefore, modulating the activation of NF-κB and inhibiting the degradation of IκB hold potential for mitigating the cytokine storm and ameliorating the severity of ARDS.

#### Notch signaling pathway

The Notch signaling pathway is well-studied for regulating cell proliferation and differentiation in respiratory system [80]. Currently, four Notch receptors (Notch 1, 2, 3, and 4) and five ligands (Jagged-1, 2 and delta-like ligand 1, 3, and 4) have been identified in mammals. Upon activation, the Notch intracellular domain is released and translocates to the nucleus, where it activates the transcription of target genes such as Hairy/Enhancer of Split 1 (Hes1) [81]. Several studies have highlighted the significant role of the Notch pathway in sepsis and ARDS, particularly in promoting proinflammatory cell polarization. Notch activation in macrophages drives M1 polarization to induce inflammation, whereas the inactivation of Notch signaling typically contributes to alternatively activated (M2) macrophage polarization that alleviates inflammation [82, 83]. Li et al. [84] reported that Notch signaling is involved in T helper 17 (Th17) cell differentiation, which releases IL-17 and IL-22 to aggravate lung inflammation and neutrophil infiltration in an LPS-induced ALI model. Conversely, some research have also suggested a role for Notch signaling in anti-inflammatory responses. Lu et al. [85] found that mesenchymal stem cells activate Notch signaling, leading to the production of regulatory DCs, which inhibit inflammatory responses against LPS-induced ALI. Whether this signaling pathway facilitates or inhibits ARDS remains inconclusive.

#### Janus kinase (JAK)/STAT signaling pathway

The essential role of the JAK/STAT signaling pathway in apoptosis, differentiation, and inflammation is widely studied. The JAK family consists of four members (JAK1, 2, 3, and tyrosine kinase 2), while the STAT family comprises seven members (STAT1, 2, 3, 4, 5A, 5B, and STAT6) [86]. This pathway functions by transmitting extracellular signals from cytokines or growth factors to the nucleus, triggering the transcription of target genes [87]. JAK/STAT mediated by cytokines plays a critical role in amplifying inflammatory signals. For example, JAK/STAT pathway can amplify inflammation both in immune and non-immune cells. The activation of JAK/STAT3 by IL-6 promotes the differentiation of Th17 cells, CD8^+^ T cells, and B cells while inhibiting the development of regulatory T cells [88]. Type II-IFN-mediated JAK/STAT1 activation induces pro-inflammatory M1 phenotypes (Fig. 2c) [89]. STAT1, STAT3 and STAT5, when activated by granulocyte colony-stimulating factor (G-CSF) promote the accumulation and activation of neutrophils in the lung (Fig. 2d) [90]. Excessive activation of these immune cells leads to an unrestrained release of pro-inflammatory cytokines and chemokines, aggravating lung injury [91]. In non-immune cells, such as pulmonary endothelial cells and AECs, the IL-6/JAK/STAT3 axis induces the releasing of various inflammatory cytokines and chemokines, significantly associated with the severity of ARDS (Fig. 2b) [92].

Moreover, JAK/STAT is implicated in the differentiation of anti-inflammatory immune cells, suggesting its potential effect for relieving the inflammatory reaction on ARDS. IL-4 and IL-2 participate in Th2 differentiation, leading to the release of anti-inflammatory cytokines that counteract Th1 cells through the activation of STAT6 and STAT5, respectively [91, 93]. Activation of JAK/STAT6 triggered by Th2-related cytokines such as IL-4 and IL-13, along with the JAK1/STAT3 signaling triggered by IL-10, may promote M2 polarization. This polarization is pivotal in inflammatory resolution and lung fibroproliferative response in the late phase of ALI/ARDS (Fig. 2c) [89, 94].

Here, the function of JAK/STAT pathway is precisely controlled by diverse inflammatory cytokines. Modulating the effects of downstream JAK/STAT pathway by targeting pro-inflammatory cytokines or/and their respective receptors may have therapeutic efficacy in ARDS.

#### MAPK signaling pathway

The MAPKs are a class of serine/threonine protein kinases in cells that transmit signals through a three-tiered sequential phosphorylation cascade and induce various cellular responses [55]. MAPKs are subdivided into four distinct subfamilies, namely extracellular signal-regulated kinase (ERK) 1 and ERK2, c-Jun N-terminal kinase (JNK), p38MAPK, and ERK5 [95]. The activation of MAPK pathway can be initiated by multiple stimuli, such as growth factors and cytokines, through their interaction with specific receptors. Additionally, environmental stress and infections can directly trigger MAPK activation [96]. Recent studies have underscored the pivotal role of the MAPK pathway in the inflammatory processes in ARDS, primarily through the facilitation of the release of inflammatory cytokines and chemokines [97–99]. Besides, accumulating evidence have demonstrated that MAPK activation aggravates lung inflammation of ARDS by upregulating the activity of NLRP3 inflammasome and NF-κB in animal models [100–102]. In addition, previous studies have revealed the involvement of the MAPK pathway in eliciting tissue factor expression in endothelial cells under inflammatory stimuli such as TNF-α and C-reactive protein, which results in activating the coagulation system and fibrin deposition (Fig. 2b) [103–105]. Thus, blocking the MAPK signaling may reduce lung damage from ARDS by alleviating inflammatory response and the clotting cascade.

#### PI3K/AKT signaling pathway

The PI3K/AKT pathway is ubiquitous in cells and participates in numerous pathophysiological processes of ARDS [106]. Cell surface receptor tyrosine kinases and GPCRs recognize their ligands, activating PI3K, which in turn converts phosphatidylinositol 4,5-bisphosphate into phosphatidylinositol 3,4,5-trisphosphate to activate AKT [107]. The role of the PI3K/AKT pathway in regulating inflammation during ARDS remains controversial. Zhong et al. [108] recently reported that the mammalian target of the rapamycin (mTOR), a downstream target of PI3K/AKT, phosphorylates the downstream transcription factor HIF-1α to induce a glucose metabolic reprogramming of macrophages, resulting in NLRP3 inflammasome activation and aggravate macrophage-mediated inflammation in LPS-induced ALI model. Besides, several studies have shown that the activation of the PI3K/AKT pathway increases inflammatory cytokines by activating the downstream NF-κB signal [109–111]. However, some recent studies have drawn a different conclusion, suggesting that the activation of PI3K/AKT is related with the alleviation of lung inflammation [112, 113]. Zhong et al. [113] reported that PI3K/AKT activation inhibited downstream NF-κB and NLRP3 inflammasome to alleviate inflammation in LPS-induced ALI model. Therefore, further investigation is necessary to explore the potential positive and/or negative effects of PI3K/AKT pathway in regulating inflammation in ARDS.

#### ER stress-mediated signaling pathway

Various pathological conditions, such as sepsis, trauma, ischemia, and viral infections, can induce ER stress, defined as the accumulation of unfolded or misfolded proteins in the ER lumen [114]. Protein kinase RNA-like ER kinase (PERK), inositol requiring kinase 1α (IRE1α), and activating transcription factor 6 (ATF6) are transmembrane proteins of ER. They transduce ER stress signals induced by cellular homeostasis imbalances, initiating the unfolded protein response, which protects the cell by degrading these unfolded or misfolded proteins [115]. However, severe or prolonged ER stress has been observed to promote inflammation in ARDS by activating a series of signals, such as MAPK and NF-κB [116–118]. Ye et al. [119] reported that the phosphorylated IRE1α during mechanical ventilation activates NF-κB signaling to promote lung injury and inflammatory processes (Fig. 2b). Given its role in the inflammatory cascade, pharmacological interventions targeting ER stress might be a potential strategy for ARDS therapy.

#### Transforming growth factor-β (TGF-β)/Small mothers against decapentaplegic (Smad) signaling pathway

The TGF-β signaling pathway is well accepted to induce lung fibrosis resulting from various diseases [120]. The interaction between TGF-β and its membrane TGF-β receptor complex leads to the phosphorylation of cytoplasmic effectors Smad2/3, forming a complex with Smad4 that translocates into the nucleus to regulate gene expression [121]. It was shown earlier that the TGF-β pathway contributes to the development of ARDS through the promotion of lung permeability, impaired epithelial ion transport, and fibrosis [34, 122, 123]. In addition, TGF-β exhibits potent proinflammatory properties. As early as 1994, Shenkar et al. [124] showed that mice administered anti-TGF-β antibodies exhibited reduced pro-inflammatory cytokine levels in comparison to untreated mice in a hemorrhage-induced ALI model. Similarly, a recent study demonstrated a reduction in inflammatory cytokine levels in an ALI model after inhibiting TGF-β/Smad signaling in vitro [125]. Another proinflammatory mechanism is that TGF-β activates MAPK and NF-κB in a Smad-independent pathway, which can occur in M1 phenotype transformation to induce inflammation (Fig. 2c) [89, 126]. In summary, active TGF-β signaling plays a critical role in ARDS, making it a potential therapeutic target.

#### TNF-α signaling pathway

TNF-α is a key cytokine involved in initiating and perpetuating inflammation in ARDS, produced by various cells in response to inflammatory stimuli [127]. TNF exerts its cellular effects through two cell surface receptors, TNF-receptor (TNFR) 1 and TNFR2. Binding of TNF-α to TNFR1 recruits adaptor proteins, including TNFR-associated death domain (TRADD) protein and TNFR–associated factor (TRAF) 2, activating NF-κB, MAPK, and activator protein-1 (AP-1) (Fig. 2b). These activated signals subsequently increase the expression of TNF-α to amplify the inflammatory effects [128]. The recent documentation of TNF-α's proinflammatory role in animal models of ALI/ARDS induced by LPS and severe acute pancreatitis suggests that targeting TNF-α could be an attractive therapeutic approach for ARDS [127, 129, 130].

### Increased endothelium and epithelium permeability

Emerging evidence has suggested that different modalities of cell death, such as necrosis, apoptosis, necroptosis, ferroptosis, and pyroptosis, coexist in the endothelium and epithelium of lung during ARDS, leading to barrier dysfunction and pulmonary edema. Besides, the disruption of intercellular junctions and cytoskeleton reorganization is required for the loss of alveolar-capillary barrier integrity. These events ultimately lead to the accumulation of leaking fluid and proteins in alveolar spaces.

#### Alveolar epithelial and pulmonary endothelial cell death

Apoptosis has been widely demonstrated to cause the injury of endothelium and epithelium in the lung during ARDS. This programmed type of cell death can be triggered by extrinsic or intrinsic apoptosis pathways. Several well-studied signaling pathways led by death receptors have been implicated to mediate extrinsic apoptosis, which includes the Fas/Fas ligand (FasL), TNF-α/TNFR1 and TNF-related apoptosis-inducing ligand (TRAIL)/TNF-related apoptosis-inducing ligand receptor (TRAILR) [131]. Many studies have supported the significant role of Fas/FasL signaling in epithelial apoptosis in ARDS, with elevated concentrations of Fas and FasL detected in the BALF of ARDS patients [132]. In vitro experiments demonstrated that BALF from ARDS patients induced apoptosis in a lung epithelial cell line, which could be reversed by blocking Fas/FasL signaling [133]. Besides, multiple animal studies have pointed out the role of Fas/FasL in inducing AEC apoptosis and lung edema during ALI/ARDS [134–136]. In addition, TNF-α/TNFR1-mediated apoptosis may contribute to endothelial injury in ARDS. Hamacher et al. [137] demonstrated that BALF from ARDS patients exhibited cytotoxicity towards human lung microvascular endothelial cells. This cytotoxic activity was effectively inhibited by neutralizing TNF-α antibodies. Contradictory findings regarding the role of TNF-α/TNFR1 in mediating alveolar epithelial apoptosis have complicated the understanding of ARDS. A previous in vivo study demonstrated that intratracheal TNF-α instillation did not dramatically affect the early apoptotic cell death in lung after LPS exposure [138]. While Sun et al. [139] recently found that TNF-α significantly enhances IFN-β-mediated apoptosis of airway epithelial cells in vitro. The involvement of TRAIL/TRAILR signaling in apoptosis and lung barrier dysfunction during ARDS has also been described [140]. Previous reports have identified type I-IFNs as potent inducers of TRAIL in AMs. The substantial release of TRAIL from AMs upon type I-IFN stimulation may lead to apoptosis in alveolar epithelium [141]. ARDS is also associated with the intrinsic apoptosis, which occurs due to increased permeability of the mitochondrial outer membrane, known as mitochondrial-dependent apoptosis. This form of apoptosis can be induced by various stimuli, such as elastase, ROS and LPS [33, 142]. Additionally, dynamin-related protein 1 (Drp1), a cytoplasmatic GTPases, has been proven to trigger mitochondrial-dependent apoptosis by inducing mitochondrial fission in AECs recently [143, 144]. In addition to the extrinsic or intrinsic apoptosis pathways, several other signaling pathways play a role in regulating apoptosis in ARDS. Apoptosis signal-regulating kinase 1 (ASK1), a member of MAPK kinase kinase kinase family, is ubiquitously expressed in various cell types. When the cells are exposed to inflammatory factors, ASK1 becomes activated to phosphorylate JNK and further induces cell apoptosis [145]. ASK1/JNK-mediated apoptosis in alveolar epithelium and endothelium has already been reported in various ALI models [145–147]. In contrast, the PI3K/AKT pathway exerts a protective role in resisting apoptosis by inactivating proapoptotic proteins, with its activation partially dependent on the binding to vascular endothelial growth factor (VEGF) [142, 148]. However, this protective pathway is downregulated during ARDS, partially attributed to decreased VEGF expression in injured epithelial cells, leading to aggravating alveolar-capillary injury [149, 150].

Necroptosis is another cell-destruction procedure that has been implicated in inducing endothelial/epithelial injury in ARDS. Necroptosis is initiated by various receptors (e.g., Fas, TNFR, TLRs), inflammatory cytokines and mitochondrial dysfunction. Subsequently, receptor-interacting protein kinase (RIPK) 1 and/or RIPK3 are recruited, leading to the activation of mixed lineage kinase domain-like protein (MLKL), which damages cell membrane integrity and induces necroptosis [151, 152]. Various ALI/ARDS preclinical models have recently demonstrated evidence of necroptosis in epithelial and/or endothelial barrier dysfunction, as assessed by RIPK and MLKL measurements [153–156]. Besides, a large ICU cohort study has implicated RIPK3 in the development of VILI. Subsequent animal experiments have indicated the importance of necroptotic function of RIPK3, evident in the protective effect observed in RIPK3 knockout mice, whereas MLKL knockout mice remained unaffected by VILI [157]. Moreover, lung autopsy of COVID-19 ARDS patients has found that angiopoietin (Ang) 2 levels are correlated with necrotic lung endothelial cell death, as shown by a linear correlation between levels of Ang2 and RIPK3 [158]. Based on these studies, the use of inhibitors targeting the necroptosis pathways involving RIPK and MLKL shows promise as a potential therapy for ARDS.

Autophagy, a catabolic process that degrades cytoplasmic components to maintain cell homeostasis, can have either beneficial or injurious effects in response to different stimuli [159]. While autophagy is an adaptive process, excessive autophagy can lead to cell death. Nonetheless, there is a paradoxical role of autophagy in mediating alveolar-capillary barrier function in ARDS. In previous research, H5N1 infection induced autophagy of AEC via inhibition of PI3K/AKT/mTOR1 pathway [160]. Besides, exposure to LPS was reported to induce autophagic death in human alveolar epithelial cell death via the activation of the PERK pathway upon ER stress in vitro [161]. However, a recent study suggests that LPS-induced autophagy decreases cell death in mouse lung epithelium [162]. These discrepant findings may be attributed to variations in experimental conditions, highlighting the intricate role of autophagy in the pathogenesis of ARDS. Thus, there is still high research value on the effect of autophagy in this field.

Differing from other forms of cell death, pyroptosis is an inflammatory programmed cell death induced by various pathological stimuli or microbial infections, accompanied by release of inflammatory cytokines [163]. The pyroptotic pathway comprises the canonical pathway, which is mediated by caspase-1 and relies on inflammasome activation, as well as the non-canonical pathway associated with caspase-4/5/11. The formation of canonical inflammasomes primarily involves cytoplasmic sensors, with NLRs and AIM2 being the most common [164]. Numerous studies have indicated that pyroptosis of epithelial and endothelial cells mediated by PAMPs and DAMPs could lead to increased barrier permeability and amplification of inflammatory cascade [165–167]. Thus, we suggest that modulating specific elements within the pyroptotic pathways of epithelial and endothelial cells could potentially mitigate the development of ARDS, preserving alveolar-capillary integrity and attenuating the secretion of cytokines.

#### Intercellular junction impairment of epithelium and endothelium

The normal endothelium forms connections through intracellular tight junctions (TJs) and adherens junctions (AJs). TJs consist of transmembrane proteins, including claudins, occludins and junctional adhesion molecules (JAMs), as well as cytoplasmic ZO proteins responsible for anchoring tight junctions to actin cytoskeleton [168]. VE-cadherin serves as the primary component of AJs and establishes connections with p120 catenin, β-catenin, and α-catenin to link with the actin cytoskeleton. The stabilization of VE-cadherin is achieved through the receptor tyrosine kinase Tie2 and vascular endothelial protein tyrosine phosphatase (VE-PTP), both of which prevent its internalization and thus protect against endothelial barrier disruption [4]. The intracellular structure of alveolar epithelium is similar to endothelium, but its main component of AJs is E-cadherin. The coordinate expression and interplay of AJs, TJs and the actin cytoskeleton play a key effect in maintaining the alveolar-capillary barrier integrity.

**Fig. 3:**
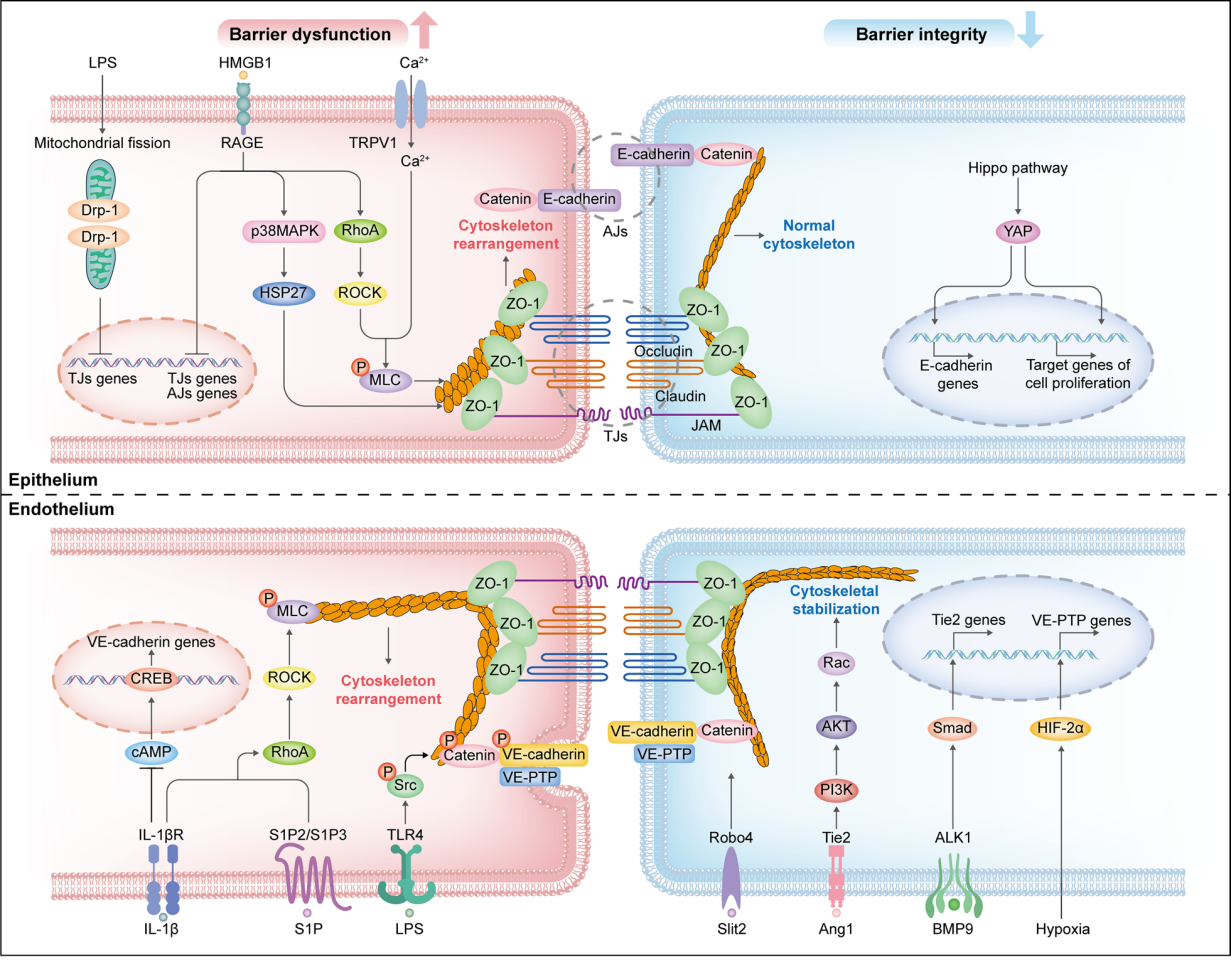
Schematic representation of the signaling pathways involved in epithelial and endothelial barrier regulation. During ARDS, the destructive signaling pathways that favor alveolar-capillary barrier disruption are predominant (left), whereas the protective signaling pathways that strengthen barrier integrity are downregulated (right), tilting the balance towards barrier disruption. Together, these result in increased alveolar-capillary permeability. AJs, adherens junctions; AKT, protein kinase B; ALK1, activin receptor-like kinase 1; Ang1, angiopoietin 1; BMP9, bone morphogenetic protein 9; cAMP, cyclic adenosine monophosphate; CREB, cyclic adenosine monophosphate response element binding; Drp1, dynamin-related protein 1; HIF-2α, hypoxia-inducible factor-2α; HMGB1, high-mobility group box 1; IL-1βR, interleukin-1β receptor; JAMs, junctional adhesion molecules; LPS, lipopolysaccharide; MLC, myosin light chain; P, phosphorylate; PI3K, phosphatidylinositol 3-kinase; RAGE, receptor for advanced glycation end products; Robo4, Roundabout 4; ROCK, Rho-associated protein kinase; S1P, sphingosine-1 phosphate; Smad, small mothers against decapentaplegic; Src, Src kinase; TJs, tight junctions; TLR, toll-like receptors; TRPV1, transient receptor potential‑vanilloid 1; VE-PTP, vascular endothelial protein tyrosine phosphatase; YAP, yes-associated protein; ZO-1, zonula occluden-1

Multiple signaling pathways have been found to downregulate the integrity of epithelial and endothelial barriers during ARDS, some of which are associated with reduced expression and distribution of AJs proteins. Xiong et al. [169] found that the disruption of the endothelial barrier by IL-1β was attributed to the downregulation of the transcription factor cyclic adenosine monophosphate (cAMP) response element binding (CREB), along with its target VE-cadherin (Fig. 3). Besides, the internalization of VE-cadherin may also lead to endothelial barrier disruption through the separation between intercellular VE-cadherin bonding. Research has provided evidence that TLR4 activation triggers Src kinase phosphorylation, subsequently leading to the phosphorylation of p120-catenin and VE-cadherin. This results in VE-cadherin internalization and increased paracellular permeability in sepsis-induced ALI model (Fig. 3) [170].

Likewise, decreased expression of TJs proteins may contribute to barrier disruption. It has been reported that Drp1-meditated mitochondria fission could induce deregulation of ZO-1 and occludins on ALI models [143]. Significantly, the interaction of RAGE and high-mobility group box 1 (HMGB1) plays a crucial role in the dysregulation of both TJs and AJs during ARDS. Studies have indicated that the HMGB1/RAGE signaling pathway downregulates the expression of VE-cadherin and E-cadherin in endothelium and epithelium, respectively, paralleled with decreased expression of TJs proteins such as occludins, claudins and ZO-1 in preclinical ARDS models (Fig. 3) [67, 171].

In addition, barrier hyperpermeability is also related to the cytoskeleton rearrangement in epithelium and endothelium, which induces actin cytoskeleton shortening, cell contraction and intercellular junction rupture [172]. The intracellular Rho GTPase family, RhoA, Rac and Cdc42, play pivotal roles as regulators of cytoskeletal rearrangement [173]. Rac1 and RhoA exhibit opposing effects: Rac1 facilitates the assembly and maintenance of AJs, whereas RhoA induces cytoskeletal contraction through the activation of Rho-associated protein kinase (ROCK) and subsequent myosin light chain (MLC) phosphorylation [174, 175]. Various inflammatory agents, such as IL-1, TGF-β, thrombin, endothelin-1 and angiotensin II, have been shown to activate the RhoA/ROCK signaling in the pathogenesis of ARDS [174]. Besides, some molecular pathways also participate in regulating RhoA/ROCK signaling. Sphingosine-1 phosphate (S1P) released by activated platelets can recognize its receptors S1P2 and S1P3 on the surface of endothelial cells, thereby inducing RhoA/ROCK-dependent barrier disruption (Fig. 3) [173]. HMGB1/RAGE has been previously studied to induce cytoskeleton rearrangement through downstream activation of p38MAPK and phosphorylation of actin-binding protein HSP27. Recently, it has been reported to activate downstream RhoA/ROCK to enhance alveolar-capillary permeability [171]. Ca^2+^ influx triggered by the activation of transient receptor potential‑vanilloid 1 channels has been reported to induce cytoskeleton rearrangement in alveolar epithelium of seawater inhalation-induced ALI model, but whether RhoA/ROCK is involved has not been elucidated (Fig. 3) [62].

Moreover, the epithelial–mesenchymal transition (EMT) is considered a pivotal phenomenon during the progression of ARDS. During this process, an epithelial cell line loses its epithelial morphology and gains mesenchymal morphology, as manifested by the downregulation of intracellular junction proteins along with the expression of profibrotic proteins such as α-smooth muscle actin. Studies have demonstrated the activation of the Wnt/β-catenin pathway in ALI/ARDS. Wnt protein released by macrophages combines to its receptor Frizzled on the membrane of alveolar epithelium, resulting in β-catenin translocation to the nucleus and subsequent regulation of various genes. This process ultimately promotes EMT and induce pulmonary fibrosis [176–178]. Interestingly, recent reports have indicated that Wnt signaling upregulated by mesenchymal cells under hypercapnia condition impairs the proliferative capacity of alveolar epithelial cells by inhibiting downstream β-catenin signaling, leading to epithelial barrier dysfunction and exacerbates pulmonary edema [179]. Thus, modulating this pathway could serve as a therapeutic strategy to alleviate fibrosis and promote lung repair after injury.

Several signaling pathways exert barrier protective functions in lung tissue, although they are generally downregulated during ARDS. Many molecular pathways within the endothelium collaborate to enhance barrier function through the stabilization and increased expression of VE-cadherin. The Ang-Tie2 signaling axis has been extensively studied as one of the pathways implicated in inducing endothelial barrier dysfunction during inflammatory diseases like sepsis and ARDS [180]. Both Ang1 and Ang2 are ligands of Tie2 but exert an opposite role in this signaling pathway by competitive binding Tie2. Activation of Tie2 by Ang1 leads to the inhibition of Src kinase, preventing the internalization of VE-cadherin [181]. Besides, the Ang1/Tie2 signaling also activates downstream PI3K/AKT to activate Rac1 kinase, leading to prevent cytoskeleton rearrangement (Fig. 3) [182]. Nevertheless, the elevated levels of Ang2 during ARDS hinder these vascular protective effects of Tie2 activation [183].

Additionally, the upregulation of Tie2 expression can provide additional protection for vascular barrier integrity by preventing the disruption of VE-cadherin junctions. In a recent study, the protective role of endogenous bone morphogenetic protein 9 (BMP9) has been demonstrated in a murine ALI model. Exogenously applied BMP9 binds to its receptor, activin receptor-like kinase 1 (ALK1) exclusively expressed in endothelial cells, leading to increase Tie expression and preventing further VE-cadherin internalization (Fig. 3). However, the protective effect of BMP9 for barrier integrity is diminished due to the cleavage of BMP9 by neutrophil-derived proteases during ARDS [184]. The Roundabout 4 (Robo4) is an endothelial-specific receptor, which prevents VE-cadherin internalization via interacting with the endothelium-derived ligand Slit2 to suppress vascular permeability (Fig. 3) [185]. Exogenously applied Slit2 N-terminal fragment has previously been demonstrated to protect mice against vascular leakage in the lung exposed to various conditions [186]. However, a recent study by Morita et al. [187] revealed that BMP9/ALK1 signaling negatively regulates the Robo4 expression. In their studies, inhibition of ALK1 in mouse COVID-19 models was found to upregulate Robo4 expression and suppress vascular permeability in the lung. Further studies are required to investigate the interaction between BMP9/ALK1 and Robo4 signaling and their exact role in ARDS.

Hypoxemia, a hallmark of ARDS in patients, has previously been implicated in the activation of HIF-2α, leading to increased VE-PTP gene expression and enhancement of the adhesive function of VE-cadherin (Fig. 3) [188]. Controlling this endogenous protective mechanism properly may be of value in patients with ARDS.

Moreover, epithelial regeneration following ARDS is recognized as crucial for improving respiratory function in the remaining lung. Recently, the Hippo/yes-associated protein (YAP) pathway has emerged as a contributor to lung repair and recovery after ALI. In the late phase of ARDS, the key effector molecule YAP transfers from the cytoplasm into nucleus to govern the expression of target genes, promoting AT II proliferation and the reassembly of epithelial AJs (Fig. 3) [189, 190].

### Impaired alveolar fluid clearance

It is well recognized that the active fluid transport is impaired in ARDS, which is associated with increased permeability of alveolar-capillary integrity and impaired AFC controlled predominately by ENaC, Na,K-ATPase, CFTR channels and aquaporins. Here we focus on the signaling pathways that mediate ion and water transport across the lung epithelium during ARDS.

**Fig. 4:**
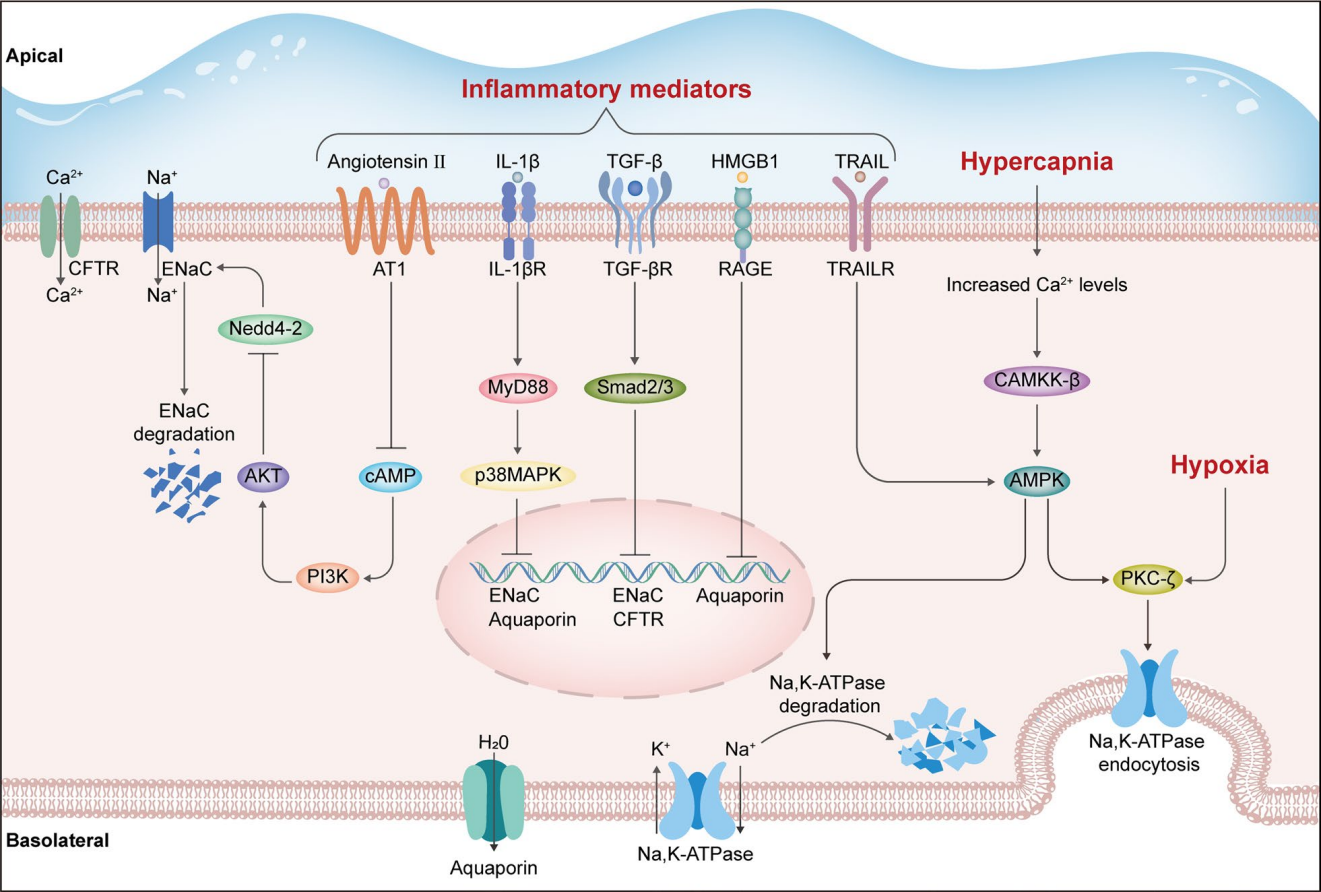
Schematic representation of the signaling pathways that impair alveolar fluid clearance during ARDS. AKT, protein kinase B; AMPK, AMP-activated protein kinase; AT 1, Angiotensin II receptor 1; CAMKK-β, Ca^2+^/calmodulin-dependent kinase kinase-β; cAMP, cyclic adenosine monophosphate; CFTR, cystic fibrosis transmembrane conductance regulator; ENaC, epithelial Na^+^ channel; HMGB1, high-mobility group box 1; IL-1β, interleukin-1β; IL-1βR, interleukin-1β receptor; MyD88, myeloid differentiation primary response gene 88; Na,K-ATPase, sodium–potassium adenosine triphosphatase; Nedd4-2, neuronal precursor cell expressed developmentally down-regulated protein4-2; p38MAPK, p38 mitogen-activated protein kinase; PI3K, phosphatidylinositol 3-kinase; PKC-ζ, protein kinase C-ζ; RAGE, receptor for advanced glycation end product; Smad, small mothers against decapentaplegic; TGF-β, transforming growth factor-β; TGF-βR, transforming growth factor-β receptor; TRAIL, TNF-related apoptosis-inducing ligand; TRAILR, TNF-related apoptosis-inducing ligand receptor

Previous studies have shown that elevated levels of proinflammatory factors during ARDS resulted in reduced expression of alveolar ion channels and impaired AFC. For example, IL-1β and LPS reportedly reduce the expression of ENaC via p38MAPK activation to decrease AFC [191, 192]. TNF-α induces declines in ENaC activity and expression through binding to TNFR1 [193]. TGF-β/Smad signaling decreases ENaC and CFTR expression, resulting in AFC failure [34, 194]. Elevated levels of angiotensin II after lung injury have been implicated to decrease ENaC expression through the inhibition of cAMP [195]. Besides, it has been suggested that TRAIL/TRAILR signaling induces the degradation of Na,K-ATPase independent of cell death pathway elicited by caspases, mediated by the cytoplasmic AMP-activated protein kinase (AMPK) [196]. Moreover, the expression of aquaporins has been shown to be downregulated through RAGE signaling and p38MAPK activation [197]. These signaling pathways strongly reveal a cross-link between inflammatory amplification and AFC impairment during ARDS (Fig. 4).

Furthermore, low oxygen or high carbon dioxide hypoxemia resulting from ventilation-perfusion mismatch and alveolar edema in ARDS can also downregulate the transportation of alveolar fluid. Hypoxia has been shown to directly induce protein kinase C-ζ (PKC-ζ) phosphorylation, leading to the endocytosis of Na,K-ATPase and subsequent reduction in AFC [198]. Similarly, elevated CO_2_ levels during hypercapnia have been demonstrated to increase intracellular Ca^2+^ concentration, which activates the Ca^2+^/calmodulin-dependent kinase kinase-β (CAMKK-β) and AMPK to phosphorylate PKC-ζ, resulting in Na,K-ATPase endocytosis (Fig. 4) [199, 200]. Similar findings are reported recently that alveolar epithelium exposed to CO_2_ activates ERK1/2 and the subsequent AMPK activation, leading to the activation of ubiquitin protein ligase neuronal precursor cell expressed developmentally down-regulated protein4-2 (Nedd4-2), which is a key molecular to drive the ubiquitination of ENaC. The activated Nedd4-2 thereby promotes alveolar edema via the induction of ENaC endocytosis [201, 202].

Conversely, there are many essential signaling pathways in enhancing AFC, suggesting their potential value for targeted reabsorption treatments of ARDS. An increasing number of studies have confirmed the beneficial effects of PI3K/AKT activation on AFC. It has been reported that the PI3K/Akt signaling pathway stimulates the serum and glucocorticoid-inducible kinase-1, which is a critical regulator of ENaC [203]. Han et al. [204] suggested that cAMP-regulated AFC enhancement by activating downstream PI3K/AKT signaling, which then phosphorylates Nedd4-2 to reduce ENaC degradation. Additionally, Magnani et al. [198] found that during prolonged hypoxia, HIF-1α signaling is activated to inhibit the endocytosis of Na,K-ATPase by causing degradation of PKC-ζ, which provides another potential therapeutic target for preserving AFC.

### ROS-mediated signaling pathways

The excessive generation of ROS is well-established to be causative in the pathogenesis and progression of ARDS. In brief, the biological origins of ROS are associated with NADPH oxidase (NOX), xanthine oxidoreductase (XOR), nitric oxide synthase (NOS), and dysfunctional mitochondria [172]. NOX family is one of the most well-known sources of cytoplasmic ROS. NOX1, NOX2, and NOX4 are members of NOX family that have been detected to produce ROS in the lung tissue [205]. In addition, uncoupling of the dimeric endothelial NOS (eNOS) can also induce oxidative injury through a dysregulated NO response, which produces peroxynitrite to induce protein nitration [172]. Similarly, mitochondrial-derived oxidative stress led by mitochondrial-derived ROS (mtROS) is important for regulation of inflammatory progression under cellular stress conditions such as inflammation, hypoxia, mechanical stretch and Ca^2+^ influx [32]. The activation of these ROS-producing pathways is concomitant in the course of inflammation, leading to the amplification of tissue damage and pulmonary edema.

During ARDS, inflammation enhances ROS production by increasing the expression and activity of ROS-producing enzymes, which in turn aggravates inflammation by initiating proinflammatory signals. For example, NF-κB is demonstrated to be activated by NOX-derived ROS in LPS-induced ALI model, resulting in the expression of inflammatory cytokines [206]. The production of ROS by NOX2 in neutrophils plays a role in TNF-α-induced NF-κB-dependent lung inflammation in mice [207]. Similarly, mtROS promotes inflammation via initiating the activation of NLRP3 inflammasome and TLR9 signaling [208]. Recently, Zeng et al. [209] found that the TLR4 activation induces NOX2 assembly in alveolar epithelium, leading to ROS-stimulated ER stress and subsequent inflammation.

ROS has also been demonstrated to contribute to the disruption of the alveolar-capillary barrier, manifested by epithelial/endothelial cell death and loss of intercellular connections. ROS acts as an upstream signal of NLRP3 inflammasome activation, leading to cell pyroptosis in both epithelium and endothelium, ultimately increasing permeability [210, 211]. Ferroptosis is a newly recognized form of programmed cell death, which is manifested by elevated ROS levels and lipid peroxidation [212]. Studies have shown that excessive ferroptosis can aggravate lung tissue damage in ALI models [213, 214]. In addition to the direct disruption of intercellular junction proteins, ROS can also trigger associated signaling pathways that negatively regulate alveolar-capillary barrier [215]. Previous studies have demonstrated that eNOS uncoupling disrupts the pulmonary endothelial barrier [216]. Rafikov et al. [217] found that peroxynitrite produced by eNOS leads to RhoA nitration, which enhances cytoskeletal rearrangement to increase endothelial permeability. Recently, it has been reported that increased NOX4-dereived ROS during ALI disrupt the endothelial barrier by activating cytosolic Ca^2+^/calmodulin-dependent protein kinase II to trigger MLC-mediated cytoskeletal contraction, leading to the aggravation of sepsis-induced ALI [218].

Besides influencing cell–cell interactions, ROS also contributes to the reduction of AFC. It is implicated that mtROS released from mitochondria under hypoxia directly activates PKC-ζ, which further induces Na,K-ATPase endocytosis and impairs AFC [219]. NOX4-mediated ROS generation triggered by upstream TGF-β/Smad signaling has been shown to promote ENaC endocytosis, resulting in reducing alveolar fluid reabsorption [34]. Considering the involvement of ROS in ARDS development and progression, targeting the enzymes responsible for ROS generation could be a promising therapeutic approach for ARDS/ALI.

The current understanding is that the protective mechanism involves the nuclear factor erythroid 2-related factor (Nrf2) pathway against oxidative stress. Nrf2 is a transcription factor that remains sequestered in the cytosol when bound to Kelch-like ECH-associated protein 1 (Keap1) under resting condition. Upon oxidative stress, free Nrf2 translocates into the nucleus to initiate the expression of antioxidative genes such as heme oxygenase (HO), superoxide dismutases, glutathione peroxidase 4 and catalase [220]. However, during ARDS, the anti-oxidative effects by Nrf2 pathway are rapidly overwhelmed by excessive ROS production, or are dysregulated in damaged tissues, leading to aggravate oxidative injury [221, 222]. The importance of Nrf2 pathway in mediating antioxidant effects has been well characterized both in vivo and in vitro models of ALI/ARDS [102, 223]. In addition to considering general Nrf2 activation, researchers have explored alternative signaling pathways as potential targets for therapy. Guo et al. [224] have shown that hyperoxia exposure induces S-glutathionylation of fatty acid binding protein (FABP) 5 in macrophages, which enhances FABP5 ability to activate PPARβ/δ and inhibit inflammation. In their study, macrophage-specific glutaredoxin 1 deficiency alleviates ALI inflammation via enhancing the levels of S-glutathionylation FABP5. Besides, Cai et al. [225] have recently revealed the important role of the Notch pathway in regulating oxidative stress. Notch1/Hes1 activation inhibits NOX4 expression, leading to attenuate ROS-mediated endothelial cell apoptosis in burn-induced ALI model. Most recently, the activation of mitochondrial uncoupling proteins UCP2, a key regulator of intracellular ROS homeostasis, has been reported to suppress ROS generation through the activation of downstream Sirtuin 3 and the antioxidant peroxisome proliferator-activated receptor gamma coactivator 1-alpha in severe acute pancreatitis-induced ALI [226]. Thus, targeting these molecular pathways may present a promising therapeutic strategy for alleviating oxidative injury in ARDS.

## Emerging pharmacologic therapies for ARDS

Pharmacotherapeutic approaches for ARDS have been attempted and tested for more than 50 years. Nonetheless, effective and targeted treatments for the disease remain elusive. Here, we will discuss the prospective pharmaceutical interventions associated with the pathophysiological and molecular targets, along with their effects on the relevant signal transduction pathways involved in ARDS management.

### Pharmacologic therapies for ARDS

#### Therapeutic agents potentially targeting anti-inflammation

Numerous pharmacological agents have been reported to appear promising in ALI/ARDS for mitigating inflammatory responses. As PRRs play an important role in provoking inflammatory injury, therapeutic strategies focused on targeting these receptors have emerged (Table 2). Recently, it has been found that Cirsilineol [227], Diacerein [115], and Taurine [228] ameliorate inflammatory injury via the TLR4/NF-κB signaling pathway after ALI. Glycyrrhizin is reported to reduce LPS-induced ALI by TLR2 signaling inhibition [229]. Of note, a recent study showed that Omeprazole encapsulated by applying nanostructured lipid carriers effectively target lung macrophages and inhibit multiple TLR pathways, including TLR3, TLR4, and TLR7/8 in a murine model of ALI, supplying a new therapy for clinical needs of ARDS [230].

**Table 2:** Recent therapeutic agents and target pathways in ARDS

Therapeutic agents/effect	Target pathways	Preclinical studies	Clinical trials
Anti-inflammation			
Glycyrrhizin	TLR2	[229]	None
Omeprazole	TLR3/4/7/8	[230]	None
Cirsilineol	TLR4/NF-κB	[227]	None
Diacerein	TLR4/NF-κB	[115]	None
Taurine	TLR4/NF-κB	[228]	None
Glibenclamide	NLRP3 inflammasome	[231]	None
Tetracycline	NLRP3 inflammasome	[232]	NCT04079426
4-hydroxynonena	NLRP3 inflammasome	[233]	None
Dexmedetomidine	HMGB1	[234]	NCT04358627
Calycosin	HMGB1	[235]	None
Tanreqing	HMGB1	[236]	None
RU.521	cGAS-STING	[237]	None
H-151	cGAS-STING	[238]	None
Chrysosplenol D	MAPK/NF-κB	[239]	None
Daphnetin	MAPK/NF-κB	[165]	None
Inula japonica	MAPK/NF-κB	[102]	None
BAP31	MyD88/NF-κB	[240]	None
Schisandrin B	MyD88/NF-κB	[241]	None
Loganin	NF-κB/NLRP3 inflammasome	[242]	None
Dapagliflozin	NF-κB/NLRP3 inflammasome	[243]	None
Hederasaponin C	NF-κB/NLRP3 inflammasome	[244]	None
Artesunate	NF-κB/NLRP3 inflammasome	[245]	None
Syringaresinol	NF-κB/NLRP3 inflammasome	[246]	None
Simvastatin	NF-κB	[247]	ISRCTN88244364
Sivelestat	NF-κB	[248]	NCT04973670
Methotrexate	JAK2/STAT3	[254]	None
Pterostilbene	JAK2/STAT3	[255]	None
Berberine	JAK2/STAT3	[256]	None
Nicotinamide	MAPK	[101]	None
Irigenin	MAPK	[259]	None
β-Caryophyllene	MAPK	[260]	None
Dilmapimod	MAPK	-	NCT00996840
Vitamin-D	ER stress	[262]	NCT03096314
AEDS	ER stress	[263]	None
Protecting alveolar-capillary barrier function			
Oxypeucedanin	PI3K/AKT, NF-κB, MAPK	[265]	None
Forsythiae	TLR4/MAPK/NF-κB	[266]	None
AL-1	NLRP3 inflammasome	[267]	None
Pazopanib	MAP3K2 and MAP3K3	[268]	None
Ruscogenin	TLR4/Src	[170]	None
Verdiperstat	Myeloperoxidase/μ-calpain/β-catenin	[269]	None
Blebbistatin	Wnt5a/β-catenin	[270]	None
Necrostatin-1	RIPK1	[273]	None
Aloperine	RIPK1/RIPK3	[274]	None
AFC promotion			
AP301	ENaC	[281]	NCT03567577
RCTR1	cAMP/PI3K/AKT	[285]	None
MCTR1	cAMP/PI3K/AKT	[204]	None
Aldosterone	cAMP/PI3K/AKT	[203]	None
Ursodeoxycholic acid	cAMP/PI3K/AKT	[286]	None
Anti-oxidation			
Panaxydol	Nrf2/HO-1	[291]	None
Melatonin	Nrf2/HO-1	[292]	None
Sitagliptin	Nrf2/HO-1	[293]	None
Quercetin	NOX2	[294]	None
Apocynin	NOX2	[295]	None
VAS2870	NOX2	[215]	None
G137831	NOX1/4	[296]	None

For NLRs family, most of the research have focused on targeting the NLRP3 inflammasome to reduce the release of cytokines. Preclinical studies have shown that Glibenclamide [231], Tetracycline [232], and 4-hydroxynonena (an endogenous product of lipid peroxidation) [233] inhibit NLRP3 inflammasome activation independently of NF-κB signaling. Besides, an observational study for Tetracycline to investigate inflammasome activation in clinical ARDS is recruiting (NCT04079426). Similarly, limiting RAGE-mediated inflammation may be beneficial in ARDS treatment. It is reported that Dexmedetomidine [234], Calycosin [235], and Tanreqing [236] alleviate inflammation mediated by RAGE and TLR4 receptors via inhibiting HMGB1 signaling. Notably, a clinical trial of Dexmedetomidine for ARDS in critical care COVID-19 patients is under investigation (NCT04358627). The targeted inhibition of the cGAS-STING pathway is also a valuable idea, and several small-molecule inhibitors of cGAS-STING pathway, such as H-151 and RU.521 have been proven to alleviate lung injury in ALI models [237, 238].

The central role of NF-κB in proinflammatory signaling pathways makes it an attractive target for pharmaceutical intervention. Considering that NF-κB integrates numerous upstream signals, many targets indirectly inhibit NF-κB-mediated inflammation via modulating its upstream signaling. It has been shown that Chrysosplenol D [239], Daphnetin [165] and Inula japonica [102] ameliorate acute lung inflammation by suppressing the MAPK-mediated NF-κB pathway. The molecular BAP31 as well as Schisandrin B have presented therapeutic value by targeting MyD88 to reduce NF-κB activation in ALI mice [240, 241]. Moreover, there are a variety of targets that inhibit NF-κB signaling to negatively regulate NLRP3 inflammasome, such as Loganin [242], Dapagliflozin [243], Hederasaponin C [244], Artesunate [245], Syringaresinol [246], all of which have been studied in preclinical models of ALI/ARDS. Of note, the neutrophil elastase inhibitor Sivelestat and Simvastatin, two promising therapeutic drugs for treating ALI/ARDS, were also proven to target NF-κB inhibition in LPS-induced ALI [247, 248]. The administration of Sivelestat to ARDS patients has been confirmed to provide a 90-day mortality advantage by a retrospective study [249]. A phase III trial is in progress to assess the impact of Sivelestat on ARDS patients with sepsis (NCT04973670). For Simvastatin, a phase IIb randomized trial of Simvastatin therapy in ARDS patients did not yield improvements in clinical outcomes (ISRCTN88244364) [250]. However, a secondary analysis of this Simvastatin trial suggested that patients with hyperinflammatory subphenotypes who received Simvastatin exhibited lower 28-day mortality [251].

Given the promising efficacy of JAK inhibitors in the treatment of COVID-19-induced ARDS, the potential therapeutic effect by targeting JAK/STAT signaling pathway in ARDS has been revealed and may become a new strategy for treating other types of ARDS. Multiple clinical trials of JAK inhibitors including Baricitinib, Nezulcitinib, Pacritinib, Ruxolitinib, and Tofacitinib are completed or under investigation, and some published results have shown their safety and efficacy in COVID-19 patients [252]. Notably, Baricitinib has received approval for the treatment of hospitalized adults with COVID-19 who require supplemental oxygen, noninvasive or invasive mechanical ventilation, or extracorporeal membrane oxygenation [253]. Besides, numerous preclinical animal studies have demonstrated the anti-inflammatory effects of JAK2/STAT3 inhibition in other etiologies-induced ARDS (Table 2) [254–256]. In addition, targeting the potent activator of JAK/STAT signaling, such as the IL-6 inhibitor Tocilizumab, has also shown beneficial effects in severe COVID-19 patients [257, 258]. Given the diverse etiology of ARDS, it is now imperative to conduct additional clinical trials for IL-6 inhibitors in non-COVID-19 ARDS patients.

Inactivation of MAPK signaling is also demonstrated to contribute to the anti-inflammatory effects in ARDS treatment. Nicotinamide [101], Irigenin [259] and β-Caryophyllene [260] have been recently found to inhibit MAPK signaling and reduce expression of inflammatory factors in ALI model. Particularly, Dilmapimod, a specific p38MAPK inhibitor, has been reported to have a satisfactory safety profile in trauma patients at risk of developing ARDS and to reduce the concentration of pro-inflammatory cytokines [261].

Recently, the aqueous extract of Descuraniae Semen (AEDS) as well as Vitamin-D are reported to possess an anti-inflammatory effect in preclinical ALI models via targeting the ER stress markers IRE1α and ATF6, respectively, offering new insights for the treatment of ALI/ARDS [262, 263]. However, a largest published randomised controlled trial showed no benefit of vitamin D on 90-day mortality in critically ill patients at high risk for ARDS [264]. With the development of more anti-inflammatory drugs, there is an increasing demand for additional high-quality clinical trials to confirm the therapeutic effects of these drugs in human ALI/ARDS patients.

#### Therapeutic agents potentially protecting the alveolar-capillary barrier

An alternative therapeutic strategy under consideration is to promote alveolar-capillary barrier function via enhancing intercellular junctions and diminishing pulmonary epithelial and endothelial cell injury. Oxypeucedanin [265], Forsythiae [266] and the andrographolide derivative AL-1 [267] have been found to contribute to the maintenance of alveolar-capillary integrity by increasing the expression of TJs proteins in ALI model. Pazopanib is shown to increase pulmonary barrier function via specifically inhibiting MAP3K2 and MAP3K3 phosphorylation in neutrophils, which leads to moderately ROS production that activates the Rac1-mediated protective effects in alveolar-capillary barrier in animal models. It also exhibits benefits in reducing lung edema in preliminary human study of five pairs of lung transplantation patients [268]. In recent years, some promising drugs have shown potential clinical benefits for treating ARDS through the protection of pulmonary endothelium. Research has shown that Ruscogenin upregulates the expression of p120 catenin and VE-cadherin via inactivating the TLR4/Src signaling in mice with sepsis-induced ALI [170]. Verdiperstat, a myeloperoxidase inhibitor, enhances VE-cadherin stability by reducing the activation of myeloperoxidase/μ-calpain/β-catenin signaling pathway on experimental ARDS in rats [269]. Blebbistatin is a myosin II inhibitor that resists pulmonary endothelial barrier dysfunction in mice. Results indicated that Blebbistatin downregulates the Wnt5a/β-catenin pathway and exerts a protective effect on lung injury [270]. In addition, certain compounds or drugs have been reported to mitigate alveolar epithelial and pulmonary endothelial cell death in preclinical models of ARDS, of which safety and efficacy remain to be further examined in clinical studies [153, 258, 271]. Of note, multiple RIPK1 inhibitors that suppress necroptosis have progressed beyond Phase I safety trials in human clinical studies for other inflammatory conditions like ulcerative colitis and rheumatoid arthritis [272]. Besides, Necrostatin-1 [273] and Aloperine [274] have obtained promising results in ARDS experimental models by reducing necroptosis and inflammation. Considering the vital role of necroptosis in the type of alveolar epithelial death in ARDS, these RIPK inhibitors merit further investigation in clinical trials [275].

#### Therapeutic agents potentially enhancing AFC

It is accepted that enhancement of AFC is pivotal for patient survival, thus the potentially effective drugs that promote excessive fluid clearance during ARDS merit investigation. β-adrenergic agonist is a commonly studied agent to improve AFC in animal models, mechanistically acting by increasing intracellular cAMP levels to increase the expression of ion transport channels [276]. Previous clinical trials involving Salbutamol for ARDS patients indicated poor tolerance and the potential to worsen mortality [277, 278]. However, a prospective study showed that inhalation of Formoterol and Budesonide reduced the incidence of ARDS [279]. A recent study reported similar results that inhaled salbutamol as monotherapy or combined with corticosteroids reduced the incidence of ARDS development among hospitalized patients [280]. Thus, this evidence may suggest the potential protective benefit of prior administration of β-adrenergic agonists in preventing ARDS. A synthetic peptide agent (a.k.a. AP301, solnatide) was shown to markedly reduce pulmonary edema by activating sodium channels in animal models of ARDS [281, 282]. A small phase 2 randomized blinded trial suggested that inhaled AP301 every 12 h for 7 days was shown to decrease pulmonary edema and reduce ventilation pressures in patients with ARDS [283]. Another trial testing AP301 in patients with moderate–severe ARDS is currently enrolling (NCT03567577).

Moreover, the important role of the macrophage-derived specialized pro-resolving mediators (SPMs) in promoting AFC during ARDS has been gradually recognized [284]. For example, the resolvin conjugates in tissue regeneration 1 [285] as well as the maresin conjugates in tissue regeneration 1 [204] belong to SPMs, which have been recently implicated to upregulate ENaC and Na,K-ATPase by activating the cAMP/PI3K/AKT signaling pathways, and results in alleviating pulmonary edema in preclinical ARDS models. However, there is a lack of significant clinical studies of either administered exogenously or induction of endogenous SPMs in ARDS patients. Besides, Ursodeoxycholic acid [286] and Aldosterone [203] have been proven to exert therapeutic effects in mitigating LPS-induced pulmonary edema in animal models by modulating the cAMP/PI3K/AKT pathway. Further researches into the efficacy and safety of these drugs in ARDS patients are required.

#### Therapeutic agents potentially attenuating oxidative injuries

Considering the crucial role of oxidative injury in ARDS pathogenesis, therapeutic targets for suppressing oxidative stress have aroused considerable attention. A variety of antioxidant therapies including vitamin C supplementation or N-acetylcysteine administration, have been applied to ARDS patients. Regarding vitamin C, a phase 2 clinical trial involving 167 patients with ARDS and sepsis found that high-dose vitamin C infusion, when compared to a placebo, did not significantly reduce organ failure or improve inflammatory biomarkers, but improved the secondary outcomes of 28-day mortality, ICU-free days, and hospital-free days [287]. N-acetylcysteine is well-known for its mucolytic effect and robust antioxidant activity. However, the usefulness of N-acetylcysteine in ARDS patients is controversial [288, 289]. Recently, its potential to inhibit the progression of COVID-19 has rendered it a highly promising therapy for the disease [290].

Moreover, the Nrf2 pathway plays a crucial role in protection against oxidative lung injuries during ARDS, and thus antioxidants activating Nrf2 pathway may be an effective intervention. It has been found that Panaxydol [291], Melatonin [292] and Sitagliptin [293] act on Nrf2 pathway to increase the expression of antioxidants in lung tissue, leading to alleviate oxidative injury in animal ARDS models. But further clinical trials are certainly required to determine its precise efficacy in protecting against ARDS. In addition, targeting the enzymes responsible for ROS generation might offer a promising therapeutic approach for ARDS. Pharmacological inhibitors of NOX2, such as Quercetin [294], Apocynin [295] and VAS2870 [215], as well as NOX1/4 inhibitor G137831 [296], have been shown to protect lung tissue damage induced by oxidative stress during ARDS. However, these have only undergone preclinical studies and evidence in human is still required.

### MicroRNAs in ARDS

In recent times, there has been a growing focus on the involvement of microRNAs (miRNAs) in ARDS. MiRNAs are a category of small noncoding RNAs that modulate gene expression by either inhibiting the translation of target mRNAs or facilitating the early degradation of complementary mRNAs [106]. Multiple results from preclinical studies have indicated that miRNAs may play pivotal roles in the pathophysiology of ARDS by targeting specific genes to regulate the signaling pathways. These regulatory effects extend to cellular, receptor, signaling pathways, and gene transcription levels [297]. For instance, Xu et al. [298] found that increased miR-199a-3p exacerbates LPS-induced ARDS via silencing PAK4 expression in AMS, resulting in the release of pro-inflammatory autophagosomes and cytokines in mice, all of which can be reversed by miR-199a-3p inhibitors. Yang et al. [299] observed that miR-16 overexpression mitigated LPS-induced ALI in mice by inhibiting TLR4 expression and subsequently downregulating the TLR4/NF-κB signaling pathways in mice. Furthermore, miRNA localization is a critical factor influencing its function. Extracellular miR146a-5p has been reported to trigger TLR7-dependent inflammation and endothelial barrier disruption while also exerting intracellular negative regulation of TLR signaling by targeting IRAK1 and TRAF6 expression [300, 301]. Recent research on miRNAs and their roles in preclinical ALI/ARDS models have been summarized in the table [298, 302–323] (Table 3).

**Table 3:** The potential therapeutic miRNAs in ARDS

MicroRNA/function	Target genes	Signaling pathways	Roles	References
Adverse				
miR-92a-3p	PTEN	NF-κB	Induce AMs activation	[302]
miR-155-5p	SOCS5	JAK2/STAT3	Induce inflammation and apoptosis	[303]
miR-210-3p	ATG7		Induce inflammation and apoptosis	[304]
mir -146a-3p	SIRT1	NF-κB	Induce inflammation, apoptosis and oxidative stress	[305]
miR-1224-5p	PPAR-γ	PPAR-γ/AMPKα	Induce inflammation and oxidative stress	[306]
miR-23a-5p	HSP20	ASK1	Induce inflammation and oxidative stress	[307]
miR-762	SIRT7	NF-κB	Induce inflammation and oxidative stress	[308]
miR-30d-5p	SOCS1, SIRT1	NF-κB	Induce M1 macrophage polarization	[309]
miR-221-5p	JNK2	NLRP3 inflammasome	Induce mitochondrial dysfunction and inflammation	[310]
miR-199a-3p	PAK4	PAK4/Rab8a	Upregulate AM-mediated inflammation	[298]
Protective				
miR-146b-5p	TRAF6	NF-κB	Alleviate apoptosis	[311]
miR-450b-5p	HMGB1	TLR4, RAGE	Alleviate apoptosis	[312]
miR-95-5p	JAK2	JAK2/STAT3	Alleviate apoptosis	[313]
miR-216a	JAK2	JAK2/STAT3, NF-κB	Alleviate inflammation	[314]
miR-574-5p	HMGB1	NF-κB, NLRP3 inflammasome	Alleviate inflammation	[315]
miR-155-5p	IL-17RB, IL-18R1, IL-22RA2	NF-κB	Alleviate inflammation	[316]
miR-138-5p	NLRP3	NLRP3 inflammasome	Alleviate inflammation	[317]
miR-182	GGPPS1		Alleviate inflammation	[318]
miR-494-3p	CMPK2	NLRP3 inflammasome	Alleviate pyroptosis	[319]
miR-150		MAPK	Alleviate inflammation, pulmonary edema and enhance epithelial integrity	[320]
miR-27a	PTEN	AKT	Alleviate inflammation and promote cell proliferation	[321]
miR-138-5p	EZH2		Inhibit M1 macrophage polarization	[322]
miR-377-3p	mTOR	mTOR	Stimulate protective autophagy	[323]

Of note, the influence of long non-coding RNAs (lncRNAs) and circular RNAs (circRNAs) upon microRNA function has also emerged rapidly, which regulates the downstream pathways by sequestering and competitively suppressing miRNA activity. For example, lncRNA NLRP3 promotes NLRP3 inflammasome activation by sponging miR-138-5p [317]. Similarly, circRNA N4bp1 that increased in ARDS patients has been demonstrated to facilitate M1 polarization via targeting miR-138-5p in CLP-induced ALI of mice [322]. lncRNA MINCR negatively regulates miR-146b-5p to activate the NF-κB-mediated inflammation [311].

Considering the regulatory roles of miRNAs in animal models of ARDS, the concept of employing miRNA mimics or antagomirs (synthetic miRNA inhibitors with sequences complementary to specific miRNAs) emerges as an appealing option for targeted therapy in ALI/ARDS. At present, there are few clinical trials involving miRNAs for diagnosing or treating ALI/ARDS. One ongoing clinical trial is currently recruiting participants with the expectation of validating several non-coding RNAs as new biomarkers for predicting the severity of ALI/ARDS in patients (NCT03766204). Another trial that aims to explore the expressions of miR-27b and Nrf2 in the development and treatment of ARDS patients is also recruiting (NCT04937855). Hence, there is an urgent need for clinical trials investigating the potential therapeutic targeting of miRNAs in ALI/ARDS, and the clinical application of miRNAs in ALI/ARDS deserves significant attention.

### Mesenchymal stromal cell therapy

Mesenchymal stromal cell (MSC) therapy has shown promising results in ARDS, thanks to its multi-directional differentiation potential, migration ability and immunomodulatory effects [324]. It spontaneously migrates to the injured region to influence the tissue microenvironment by secreting soluble bioactive molecules or cell–cell contact, leading to alleviate inflammation, enhancing epithelial and endothelial regeneration and improving AFC [325]. Preclinical studies have demonstrated that MSCs participate in a variety of signaling pathways associated with the pathophysiology of ARDS. Administration of human umbilical cord-derived MSCs alleviated inflammation in LPS-induced ALI of mice via downregulation of NF-κB signaling [326]. MSC-expressed jagged-1 interacts with Notch2 on mature DCs, which differentiate into regulatory DCs to negatively regulate inflammation [85]. MSC has also been demonstrated to promote barrier function and restoration of alveolar epithelium by activating Wnt/β-catenin signaling pathway in ALI mice [176]. Marrow-derived MSCs are reported to exert antioxidant effects via upregulating Nrf2/HO-1 signaling in rat models with LPS-induced ALI [327]. Inhibition of the Hippo signaling increased MSCs differentiation into ATII cells and alleviated LPS-induced ALI [328].

The safety and efficacy of transplanted MSCs for patients with ARDS have been substantiated by many clinical trials [329–332]. A phase 1/2 trial demonstrated the safety of MSC administration in ARDS patients, with the potential to reduce 28-day mortality and the requirement for ventilator support [333]. The transplantation of menstrual blood-derived MSCs has the potential to lower mortality in patients with H7N9 virus-induced ARDS, as observed during a five-year follow-up period [334]. In addition, clinical trials are currently investigating the therapeutic benefits of MSCs for COVID-19, which provide a promising opportunity for patients with pulmonary damage [335]. However, a recent multicenter, randomized, double-blind, placebo-controlled trial (NCT 03042143) has found that patients with moderate to severe COVID-19-related ARDS do not benefit from ORBCEL-C (CD362-enriched umbilical cord-derived MSCs), although the application of these MSC cells is considered safe [336]. For further information about the properties and functions of MSCs in ARDS, consider consulting the reviews by Fernandez-Francos et al. [337] and Qin and Zhao [325].

## Subphenotypes in ARDS and prospects for targeted therapies

While numerous preclinical studies on pharmacological treatments for ARDS have shown promise, none have yet demonstrated a significant impact on ARDS mortality in clinical trials. The potential reasons may be partially due to the heterogeneity of ARDS [338]. More homogeneous subgroups of ARDS patients can be identified based on physiological, clinical, and biological characteristics [339]. By integrating clinical and biological characteristics, Calfee and colleagues identified a hyper-inflammatory subphenotype characterized by increased inflammation and higher mortality compared to the hypo-inflammatory subphenotype [340]. Subsequent studies have reported similar findings [341–343]. These prognostic enrichments can identify a higher likelihood of a poor outcome and may assist in making bedside healthcare decisions.

On the other hand, predictive enrichment aids in the selection of patients with a higher likelihood of positive responses to specific treatments or in the identification of patients more likely to benefit from particular interventions based on underlying mechanisms and biological characteristics [339]. Physiological and clinical phenotyping for predictive enrichment has yielded intriguing findings. For instance, Calfee CS et al. discovered elevated levels of epithelial injury biomarkers in patients with direct ARDS [344]. Additionally, some research have shown that recruitment maneuvers are less effective in primary ARDS rat models, while methylprednisolone proves to be more effective in mitigating the inflammatory response [345, 346]. While pre-randomization trials involving biologic phenotyping for predictive enrichment are infrequent in clinical practice due to the limited availability of biomarker tests, retrospective studies have demonstrated varying responses of hypo- and hyper-inflammatory phenotypes to interventions, such as positive end-expiratory pressure, fluid management strategies, and simvastatin [251, 339, 340, 342].

Given that hypo- and hyper-inflammatory phenotypes provide only a general characterization of inflammation in ARDS, it is worthwhile to identify more specific subphenotypes based on the main signaling pathways. Further evaluation of targeted treatments has the potential to enhance therapeutic responses and improve the ability to identify effective interventions. For example, Bos et al. reported elevated expression of oxidative phosphorylation genes in the “reactive” subphenotype, as identified by plasma protein biomarkers. The authors suggested further investigation of interventions targeting this pathway in patients with “reactive” subphenotype [347]. However, as summarized by Wilson JG et al., the use of metabolomics, transcriptomics, genomics, and signaling pathway characteristics for ARDS phenotyping and predictive enrichment is still in its early stages [339].

## Conclusions

ARDS is a syndrome characterized by high morbidity and mortality. Despite substantial progress has been made over the past five decades in understanding the pathogenesis and pathophysiology of ARDS, few pharmacological interventions have shown a clear mortality benefit in its therapy. Clinical treatment mainly relies on supportive care with mechanical ventilation. Therefore, there is an urgent need for novel therapeutic strategies in ARDS treatment. As recent studies have shown, the developed therapeutic strategies have taken into consideration the regulation of signaling pathways involved in the pathophysiological mechanisms of ARDS. In this review, we combed the existing evidence of molecular mechanisms in ARDS pathophysiology, involving inflammation, increased alveolar-capillary permeability, impaired AFC and oxidative stress. Moreover, we reviewed the recent promising therapeutic strategies for managing ARDS, highlighting the pathophysiological basis and the influences on cell signaling molecule expression for their use.

Of note, pharmacologic therapies that achieved promising effects in preclinical studies have often failed to show efficiency in clinical trials involving unselected ARDS populations. These outcomes can be attributed to the clinical and biological heterogeneity of ARDS patients. Secondary analysis of data from randomized controlled trials has revealed distinct responses to simvastatin treatment, fluid strategy, and positive end-expiratory pressure strategy between the hypo-inflammatory and hyper-inflammatory subphenotypes [251, 340, 342]. By employing CT imaging data and physiological characteristics, latent class analysis revealed the presence of two subphenotypes exhibiting varying responses to lung recruitment [348]. In view of these findings, it is essential to identify homogenous biological and clinical phenotypes of ARDS and to conduct further investigations into the underlying variations in molecular mechanisms among different subphenotypes. These efforts are critical for advancing more effective targeted pharmacologic therapies and achieving precision medicine.

## Data Availability

Not applicable.

## Abbreviations

AECs: Alveolar epithelial cells
AEDS: Aqueous extract of Descuraniae Semen
AFC: Alveolar fluid clearance
AIM2: Absent in melanoma 2
AJs: Adherens junctions
AKT: Protein kinase B
ALI: Acute lung injury
ALK1: Activin receptor-like kinase 1
AMPK: AMP-activated protein kinase
AMs: Alveolar macrophages
Ang: Angiopoietin
AP-1: Activator protein-1
ARDS: Acute respiratory distress syndrome
ASC: Apoptosis-associated speck-like protein containing a CARD
ASK1: Apoptosis signal-regulating kinase 1
ATF6: Activating transcription factor 6
BALF: Bronchoalveolar lavage fluid
BAP31: B-cell receptor associated protein 31
BMP9: Bone morphogenetic protein 9
CAMKK-β: Calmodulin-dependent kinase kinase-β
cAMP: Cyclic adenosine monophosphate
CDSs: Cytoplasmic DNA sensors
CFTR: Cystic fibrosis transmembrane conductance regulator
cGAMP: Cyclic GMP-AMP
cGAS: Cyclic GMP-AMP synthase
circRNAs: Circular RNAs
COVID-19: Coronavirus disease 2019
CREB: CAMP response element binding
DAMPs: Damage-associated molecular patterns
DCs: Dendritic cells
Drp1: Dynamin-related protein 1
EMT: Epithelial–mesenchymal transition
ENaC: Epithelial Na^+^ channel
eNOS: Endothelial NOS
ER: Endoplasmic reticulum
ERK: Extracellular signal-regulated kinase
FABP: Fatty acid binding protein
FasL: Fas ligand
FPR: N-formyl peptide receptor
G-CSF: Granulocyte colony-stimulating factor
GPCRs: G-protein-coupled receptors
Hes1: Hairy/Enhancer of Split 1
HIF-1α: Hypoxia-inducible factor-1α
HIF-2α: Hypoxia-inducible factor-2α
HMGB1: High-mobility group box 1
HO: Heme oxygenase
HSP: Heat shock protein
IFNs: Type I-interferons
IL-1: Interleukin-1
IRE1α: Inositol requiring kinase 1α
IRFs: Interferon regulatory factors
IκB: NF-κB inhibitor
JAK: Janus kinase
JAMs: Junctional adhesion molecules
JNK: C-Jun N-terminal kinase
Keap1: Kelch-like ECH-associated protein 1
lncRNAs: Long non-coding RNAs
LPS: Lipopolysaccharide
MAPK: Mitogen-activated protein kinase
MAVS: Mitochondrial antiviral signaling protein
MCTR1: Maresin conjugates in tissue regeneration 1
MDA5: Melanoma differentiation-associated gene 5
miRNAs: MicroRNAs
MLC: Myosin light chain
MLKL: Mixed lineage kinase domain-like protein
MSC: Mesenchymal stromal cell
mTOR: Mammalian target of the rapamycin
mtROS: Mitochondrial-derived ROS
MyD88: Myeloid differentiation primary response gene 88
Na,K-ATPase: Sodium–potassium adenosine triphosphatase
NAIP: Neuronal apoptosis inhibitory protein
Nedd4-2: Neuronal precursor cell expressed developmentally down-regulated protein4-2
NETs: Neutrophil extracellular traps
NF-κB: Nuclear factor-κB
NLRC: Nucleotide-binding oligomerization domain like receptor subfamily C
NLRP: Nucleotide-binding domain leucine-rich repeat protein
NLRs: Nucleotide-binding leucine-rich repeat receptors
NOD: Nucleotide-binding oligomerization domain
NOS: Nitric oxide synthase
NOX: NADPH oxidase
Nrf2: Nuclear factor erythroid 2-related factor
PAMPs: Pathogen-associated molecular patterns
PERK: Protein kinase RNA-like ER kinase
PI3K: Phosphatidylinositol 3-kinase
PKC-ζ: Protein kinase C-ζ
PRRs: Pattern recognition receptors
RAGEs: Receptors for advanced glycation end products
RCTR1: Resolvin conjugates in tissue regeneration 1
RIG-I: Retinoic acid-inducible gene I
RIPK: Receptor-interacting protein kinase
RLRs: RIG-I-like receptors
Robo4: Roundabout 4
ROCK: Rho-associated protein kinase
ROS: Reactive oxygen species
S1P: Sphingosine-1 phosphate
SARS-CoV-2: Severe acute respiratory syndrome coronavirus 2
Smad: Small Mothers against Decapentaplegic
SPMs: Specialized pro-resolving mediators
STAT: Signal transducer and activator of transcription
STING: Stimulator of the interferon gene
TGF-β: Transforming growth factor-β
Th17: T helper 17 cell
TJs: Tight junctions
TLRs: Toll-like receptors
TNFR: TNF-receptor
TNF-α: Tumor necrosis factor-α
TRADD: TNFR-associated death domain
TRAF: TNFR–associated factor
TRAIL: TNF-related apoptosis-inducing ligand
TRAILR: TNF-related apoptosis-inducing ligand receptor
TRIF: Toll/interleukin-1 receptor-domain-containing adaptor-inducing interferon-β
TRP: Transient receptor potential
VE-cadherin: Vascular endothelial cadherin
VEGF: Vascular endothelial growth factor
VE-PTP: Vascular endothelial protein tyrosine phosphatase
VILI: Ventilator-induced lung injury
XOR: Xanthine oxidoreductase
YAP: Yes-associated protein
Zos: Zonula occludens
